# Teaching Acute Coronary Syndrome High-Risk ECG Interpretation and Clinical Decision-Making Through FOAMed Videos and Podcast Versus Print-Based Materials Among Emergency Care Providers: Randomized Controlled Mixed Methods Trial

**DOI:** 10.2196/87587

**Published:** 2026-09-15

**Authors:** Wiebke Turner, Klaus Fessele, Martin Fandler, Jan P Ehlers, Julia Nitsche

**Affiliations:** 1 Department of Human Medicine, Chair of Didactics and Educational Research in Healthcare, Witten/Herdecke University, Alfred-Herrhausen-Straße 50, Witten, North Rhine-Westphalia, 58448, Germany, 49 15776375303; 2 Emergency Department, Nuremberg Hospital, Nuremberg, Bavaria, Germany; 3 ADAC HEMS Academy, Weßling, Bavaria, Germany

**Keywords:** acute coronary syndrome, electrocardiogram interpretation, high-risk ECG signs, STEMI equivalents, clinical decision-making, FOAMed, digital learning, podcast-based education, video-based learning, self-directed learning, electrocardiogram, Free Open Access Medical Education

## Abstract

**Background:**

Accurate interpretation of high-risk acute coronary syndrome (ACS) electrocardiograms (ECGs) is essential for early diagnosis and timely reperfusion, yet substantial deficits persist across health care professions. Digital self-learning formats such as FOAMed (Free Open Access Medical Education) are widely used, but their effectiveness has rarely been evaluated for complex, high-risk ACS ECG patterns. Existing ECG education studies often focus on students or single professional groups and established ST-segment elevation myocardial infarction (STEMI) criteria, leaving newer guideline-recognized STEMI equivalents, selected emerging occlusion myocardial infarction (OMI)–related patterns, and interprofessional emergency care underrepresented.

**Objective:**

This study aimed to compare the effectiveness of FOAMed podcast and videos versus traditional print-based materials for teaching high-risk ACS ECG patterns and related clinical decision-making in emergency providers.

**Methods:**

We conducted a prospective, interprofessional, controlled mixed methods trial across 5 training sites in Germany. Paramedics, prehospital emergency physicians, and emergency department clinicians received either a FOAMed multimedia module or print-based materials through concealed allocation; deviations from the intended 1:1 ratio resulted from participant no-shows. The intervention consisted of a 30-minute supervised self-learning session. In total, 103 participants were allocated to FOAMed (n=45) or print-based materials (n=58). Two coprimary outcomes were assessed: ECG interpretation accuracy and text-based ACS clinical decision-making. Secondary outcomes included subjective confidence, learning experience, and exploratory qualitative free-text responses. Outcome assessment was automated and blinded; mixed ANOVA was the primary analysis. The study was not prospectively registered because it assessed educational outcomes in health care professionals rather than patient health outcomes.

**Results:**

All 103 participants completed the study. Both groups improved, with greater gains in the FOAMed group: ECG interpretation increased from 55% to 65.5% and text-based ACS clinical decision-making from 45% to 68%, versus 57% to 60% and from 47% to 63%, respectively, in the print-based group. Effect sizes were η²=0.055 for ECG interpretation and η²=0.044 for clinical decision-making. Exploratory subgroup analyses provided no evidence of differential effects across age, gender, or professional background and were likely underpowered. Qualitative responses (46 and 37 entries) provided contextual insights into perceived clarity, engagement, and practical relevance supporting the quantitative findings.

**Conclusions:**

This study is innovative in directly comparing a curated FOAMed multimedia module with selected print-based materials in an interprofessional emergency care population. It differs from existing research by focusing on subtle, emerging ischemic patterns and evaluating realistic, time-limited self-learning formats. The findings provide evidence that curated FOAMed resources can produce greater short-term improvements in ECG interpretation and text-based ACS clinical decision-making than traditional print-based materials in this setting. Although implications for clinical performance remain hypothetical, concise, high-quality digital modules may represent a practical supplement to structured continuing education in emergency care.

## Introduction

The rapid and correct interpretation of electrocardiograms (ECGs) is crucial for the initial treatment of acute coronary syndrome (ACS) [1]. In addition to classic electrocardiographic ST-segment elevation myocardial infarctions (STEMI), various guideline-recognized STEMI equivalents and selected high-risk ACS ECG patterns have been described in recent years. These include left bundle branch block and right bundle branch block in the context of ACS, hyperacute T waves, de Winter and Wellens sign, shark fin sign (as a special subtype of STEMI), Aslanger sign, semi-STEMI, and left main occlusion ECG [2-8]. Recent work further emphasizes the occlusion myocardial infarction (OMI) paradigm and the diagnostic relevance of subtle high-risk ACS ECG patterns [9,10]. Despite their clinical relevance, many of these patterns were only recently incorporated into ACS guidelines or remain insufficiently represented [1]. At the same time, several ECG phenomena closely resemble these high-risk ACS ECG patterns and are therefore referred to as “STEMI mimics” [11]. Misinterpretation of high-risk ACS ECG patterns—particularly STEMI equivalents—can lead to delayed recognition and reperfusion, whereas STEMI mimics primarily contribute to false-positive activations rather than treatment delays.

International meta-analyses and studies show that the average accuracy of ECG interpretation among medical students and physicians is only around 54% [12]. More recent analyses confirm persistent deficits across health care professions [13], with diagnostic accuracy varying by age, gender, and expertise [14]. Recent prospective data further show that even senior emergency physicians frequently miss major ECG abnormalities, with only moderate agreement compared to cardiologists [15]. Comparable deficits have been reported among emergency medical services personnel [16], and in some cases, uncertainty is so pronounced that it has been described as “ECGphobia” [17], with observational work further showing that paramedics frequently misinterpret ACS-related ECG patterns [18]. Additional systematic review evidence similarly indicates that paramedics’ ability to identify STEMI varies substantially and improves with targeted ECG training [19]. These deficits affect not only rare patterns, but also central, potentially life-threatening ECG patterns such as classic STEMI findings, which are not recognized in up to 44% of cases [13,20].

Primary contributors to these deficits include insufficient training, limited exposure, and a lack of structured ECG education [21]. Emergency medical technicians likewise report a strong need for additional ECG training [16]. Standardized training that includes both established guideline-recognized STEMI equivalents and selected emerging or previously described high-risk ACS ECG patterns is therefore essential to strengthen diagnostic competencies in emergency care.

However, access to structured continuing education is often limited by time constraints, staffing shortages, and financial pressures [22]. At the same time, digitalization offers new learning opportunities for flexible learning. Podcasts, videos, and other freely available formats in the spirit of the Free Open Access Medical Education (FOAMed) movement are increasingly being used [23], although they remain insufficiently integrated into formal curricula [23]. Digital learning formats offer time- and location-independent education, address practice-relevant content, and accommodate different learning preferences. According to cognitive load theory and the cognitive theory of multimedia learning, audiovisual formats can reduce extraneous load and support more efficient processing of complex information [24,25]. However, evidence regarding the effectiveness and implementation of digital learning in health sciences education remains heterogeneous [26]. Although numerous studies have evaluated ECG teaching interventions [27,28], no randomized studies have compared FOAMed-based self-learning formats with traditional print materials for teaching high-risk ACS ECG patterns. This represents a significant research gap: although the need for structured, practical, and efficient continuing education in emergency medicine is undisputed, robust data on which formats are most effective for which content are lacking. Previous studies have focused primarily on medical students or physicians [27,28], while evidence from emergency medical personnel remains scarce [16,18,19]. The aim of the study is to evaluate the effectiveness of digital FOAMed self-learning formats (in this case, podcast and videos) compared with traditional print materials (guidelines and textbooks) in teaching selected high-risk ACS ECG patterns. The study assesses objective knowledge gain, clinical decision-making, subjective confidence, and perceived learning experience. Additionally, differences between established and newer ECG patterns and between professional groups in emergency medicine will be analyzed. Specifically, this study addresses the following research questions:

1. What influence does 30 minutes of using various self-learning training media have on learning success?
  - Regarding interpreting ECG
  - Regarding knowledge in dealing with potential myocardial infarction patients
2. What impact does 30 minutes of using various self-training media have on the sense of safety?
3. How do participants rate the learning experience of the training media they used?
4. Which groups of people show the greatest increase in knowledge with which training medium?
5. What is the current level of knowledge of the newer high-risk ACS ECG patterns among decision-makers in emergency medicine?

## Methods

### Trial Design

**Figure 1.**
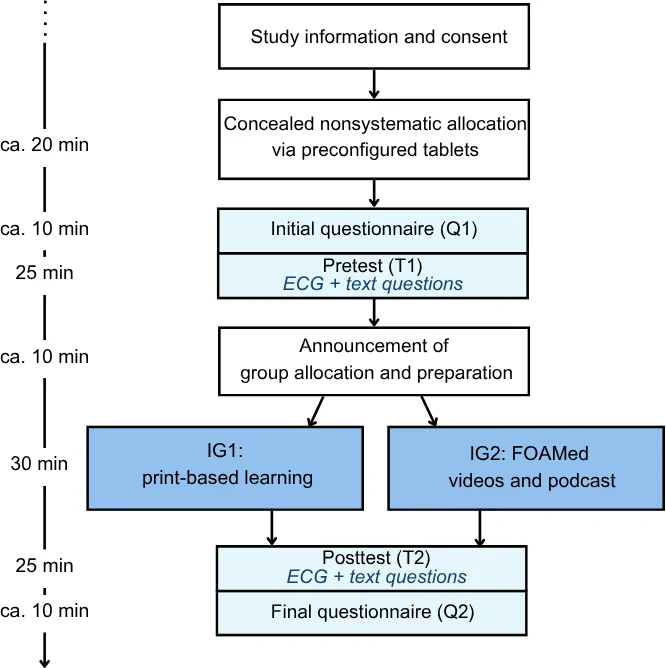
Study design of a controlled educational intervention comparing FOAMed (Free Open Access Medical Education) videos and podcast with print-based learning materials among emergency care providers in Germany in 2024. ECG: electrocardiogram; IG1: intervention group 1; IG2: intervention group 2.

This study was designed as a prospective, parallel-group controlled educational trial with 2 intervention arms and a pre-post assessment structure. The trial followed a superiority framework, comparing 2 self-directed learning formats. No changes to the trial protocol or prespecified outcomes occurred after trial commencement. No publicly accessible trial protocol or separate statistical analysis plan exists for this minimal-risk educational trial. All methodological details, including prespecified outcomes and statistical procedures, are fully reported in this paper. Figure 1 provides a visual summary of the study design, including concealed, nonsystematic allocation, timing of assessments, and the sequence of both intervention arms. The study was not prospectively registered in a primary trial registry. According to the International Committee of Medical Journal Editors (ICMJE), registration is not required for studies in which health care providers are assigned to intervention and control groups when the purpose is to assess effects on the providers themselves, such as knowledge or attitudes, rather than on the health outcomes of their patients [29]. As this study evaluated the effect of educational interventions on participants’ ECG interpretation and knowledge, without assessing patient health outcomes, prospective trial registration was therefore considered not applicable. This paper was prepared in accordance with the CONSORT (Consolidated Standards of Reporting Trials) 2025 guidelines for reporting randomized controlled trials [30].

### Participants

Participants were paramedics and physicians regularly involved in ACS-related decision-making in emergency care. Inclusion criteria were age 18‐65 years, ≥30 working hours per week, and active clinical responsibility in emergency medicine. Exclusion criteria were medical students without decision-making authority, trainees, and members of the study team.

Recruitment occurred through invitations to hospital departments and emergency services, with remaining slots publicly advertised via social media. Five in-person study events were conducted in North Rhine-Westphalia, Germany, in January 2024. Patients or members of the public were not involved in the design, conduct, reporting, or dissemination plans of this study.

Because the trial involved only self-directed educational materials without any patient contact or patient-facing procedures, patient or public involvement was not applicable.

### Trial Setting

The trial took place in classroom-based training sessions at 5 emergency-medicine teaching locations in North Rhine-Westphalia, Germany. All assessments and interventions were administered on-site under supervised conditions.

### Study Procedure

The study consisted of 2 assessment points: a pretest (Questionnaire 1/Test 1; Q1/T1) immediately before the intervention and a posttest (Questionnaire 2/Test 2; Q2/T2) directly after the 30-minute learning phase. Both assessments were completed individually on the tablets provided using the same interface and response format. After completing the pretest, the intervention instructions were presented via the projector, and all participants began the 30-minute learning phase simultaneously. The posttest followed immediately under identical supervised conditions.

### Interventions

#### Print-Based Learning

Participants in the print‑based learning group (intervention Group 1; IG1) received full, unmarked versions of established textbooks and current ACS guidelines. Materials included the following:

- *EKG-Kurs für Isabel* (Trappe and Schuster, 8th ed, 2020; ISBN 978‐3132200302)
- *EKG in der Notfallmedizin* (Schnelle, 1st ed, 2017; ISBN 978‐3943174694)
- Deutsche Gesellschaft für Kardiologie (DGK) non–ST-segment elevation acute coronary syndrome (NSTE-ACS) guideline (2020)
- DGK STEMI guideline (2017)
- European Society of Cardiology (ESC) ACS guideline (2023)
- American College of Cardiology (ACC) Chest Pain Consensus (2022)

The DGK guidelines were available in German, while the ESC guideline and the ACC Chest Pain Consensus were provided in English, reflecting their original publication languages. The textbooks were selected based on a prestudy survey of 1012 emergency care providers identifying the most used and recommended ECG resources, ensuring ecological validity and relevance for the target population. All materials were provided in full length without marked chapters. Except for the consensus paper, all print-based materials included a table of contents that participants could use to navigate relevant ACS-related sections. The core components of the print-based intervention were unrestricted access to full textbook and guideline materials and self-directed navigation of ACS-relevant content.

#### Digital Learning

Participants in the digital learning group (intervention group 2; IG2) completed a curated multimedia module consisting of the following:

- Nine short YouTube (Google LLC) videos from “Nerdfallmedizin”
- A podcast episode from “PinUpDocs” on high-risk ACS ECG patterns

All digital materials were frozen for the duration of the study, delivered via preconfigured iPads, and could be paused or replayed. The complete list of all FOAMed materials (titles, URLs, and durations) and interface screenshots is provided in Multimedia Appendix 1. The detailed learning objectives, exact sequence of multimedia elements, and the Template for Intervention Description and Replication (TIDieR)–compliant description of the intervention are fully reported in the “Methods” section.

### Delivery and Fidelity

Both interventions were delivered in a supervised, face-to-face classroom setting. Each participant had an individual desk facing the projector screen. A 30-minute timer was displayed on the projector, which was also used to introduce the intervention and explain the procedure. All participants started simultaneously, and the study lead together with an uninvolved assistant ensured a quiet environment without conversation. Both were available throughout to address technical issues but did not provide instructional guidance. No co-interventions or additional educational activities were provided during the study period.

Participants were asked in advance to bring their own Bluetooth-compatible headphones, which were paired with the tablets before the intervention started. Each participant had an individual tablet for completing the assessments; IG2 additionally used the tablet to access the multimedia module. In IG1, each participant received one copy of each listed textbook and one copy of each printed, ring-bound guideline, ensuring that all materials were individually available and not shared. Participants could take handwritten notes on a provided A5 notepad, although notes were not permitted during the posttests.

Both intervention arms addressed the same overarching topic—recognition and management of high-risk ACS ECG patterns—but the materials reflected the real-world differences between print-based and digital educational resources. The print-based group had unrestricted access to current ACS guidelines, which fully cover high-risk ECG concepts, while the textbooks reflected the publication cycle of print media. The FOAMed materials presented the same concepts in a curated multimedia format. These differences represent authentic educational ecosystems rather than methodological imbalance.

### Outcomes

#### Overview

The primary outcome was improvement in ACS-related diagnostic performance from T1 to T2, assessed through two coprimary components: (1) ECG interpretation accuracy (high-risk ACS ECG: yes, no, or unsure) and (2) text-based clinical decision-making items, consisting of true, false, or unsure statements on ACS management. Both components were administered at T1 and T2 and analyzed as coprimary outcomes. No formal trial-level success criterion was prespecified, because the two coprimary outcomes represent distinct diagnostic domains in which different learning formats could theoretically perform better.

Secondary outcomes included the following:

- Subjective confidence in ECG interpretation and ACS management
- Learning experience ratings
- Qualitative explanations for changes in perceived confidence

Clinical decision-making was assessed using text-based emergency scenarios, representing hypothetical decision-making rather than observed clinical behavior. Confidence and sense of safety were measured as self-reported perceptions and do not reflect objective clinical competence. All outcomes were prespecified. No harms or unintended effects occurred or were reported. As the intervention consisted solely of self-directed educational materials without any clinical procedures or patient contact, no adverse events were expected.

#### Test Development and Validity

All ECG and text-based items were developed under the supervision of a senior cardiologist and emergency physician (KF), who reviewed all items for clinical relevance, clarity, and unambiguous ECG morphology. ECG cases were primarily drawn from an internal case archive of a cardiology emergency department; additional cases were sourced from Dr Smith’s ECG blog, an educational resource founded by Stephen W Smith, MD, Professor of Emergency Medicine at the University of Minnesota [31], and subsequently verified for diagnostic accuracy by the supervising emergency cardiologist. Face validity was further assessed through pretesting with 8 target-group representatives (4 physicians and 4 paramedics), who evaluated comprehensibility and assigned difficulty ratings on a 1‐3 scale. Based on mean difficulty ratings, 2 parallel test forms of comparable difficulty were created for T1 and T2, and 5 items (5 ECGs and 5 text questions) were repeated across both tests to anchor difficulty. Internal consistency metrics (eg, Cronbach α) and item discrimination indices were not calculated because the instrument was intentionally designed as a heterogeneous, content-validated assessment combining ECG pattern recognition and guideline-based text questions, which measure distinct constructs and do not meet the psychometric assumptions required for such analyses. Sample ECG items and text questions are provided in Multimedia Appendix 2 to support transparency and face and content validity.

### Methodological Quality Assessment (Medical Education Research Study Quality Instrument)

The methodological quality of the quantitative component of this study was evaluated using the Medical Education Research Study Quality Instrument (MERSQI) [32]. The scoring was performed by 2 independent reviewers from the Chair of Didactics and Educational Research in Healthcare who were not involved in the study. Both reviewers first scored all 6 MERSQI domains independently and subsequently discussed 2 minor discrepancies before reaching full consensus. Domain-level scores and justifications are provided in the MERSQI checklist (Checklist 1). The MERSQI score applies exclusively to the quantitative part of the study, as the instrument is not designed to evaluate qualitative components.

### Sample Size

An a priori power analysis (G*Power 3.1; Faul, Erdfelder, Lang, and Buchner; mixed ANOVA; Cohen *f*=0.25; α=.05; power=0.80) indicated a required sample size of 126 participants. No interim analyses or stopping guidelines were planned.

### Allocation Procedure: Sequence Generation

Allocation was achieved through practically random distribution of preconfigured tablets. Half of the tablets contained the FOAMed multimedia module, while the other half contained only the assessment interface. Tablets were prepared manually before participant arrival; no computer-generated random sequence was used.

### Allocation Concealment

Tablets were placed nonsystematically on tables, and all devices displayed identical locked screens, preventing visibility of group assignment. Neither participants nor study personnel could infer allocation prior to unlocking the devices.

### Implementation

An uninformed assistant distributed the tablets, and participants freely chose seats and generated an individual study code. Because the tablets were indistinguishable and group assignment became visible only upon unlocking, allocation was fully concealed. Participants assigned to the print-based group received the printed textbooks and guidelines only after unlocking their tablet.

The unequal final group sizes (58 vs 45) resulted from participant no-shows: preconfigured tablets that were not unlocked did not contribute to the realized allocation. Seat choice did not influence allocation because tablet content could not be inferred from appearance or placement, and free seating therefore did not introduce selection bias.

### Blinding

Blinding of participants and study personnel was not feasible due to the nature of the educational interventions. Outcome assessment was automated and identical across groups, minimizing assessor bias.

### Statistical Methods (Quantitative Analysis)

#### Overview

Descriptive statistics were calculated in Microsoft Excel. Primary and secondary outcomes were analyzed using mixed ANOVA (between-subject factor: group; within-subject factor: time). Mixed ANOVA served as the primary analysis, and no additional mixed-effects modeling was required due to the complete paired dataset. All point estimates are reported with 95% CIs. No statistical adjustments for multiple comparisons were applied. Secondary outcomes and subgroup analyses are therefore interpreted as exploratory and likely underpowered. Trichotomous ECG and text-based responses (correct, incorrect, or unsure) were dichotomized into correct versus incorrect, with “unsure” coded as incorrect.

Assumption checks for the mixed ANOVA were conducted. Normality of residuals was assessed using Shapiro-Wilk tests, homogeneity of variances using Levene test, and equality of covariance matrices using the Box M test; all assumptions were sufficiently met. Because the within-subject factor included only 2 time points, sphericity was automatically satisfied. Percentage scores were treated as continuous variables, which is considered acceptable for ANOVA when distributions are not severely skewed and sample sizes are adequate. Baseline comparability between groups was confirmed by similar T1 means (Table 1). As a sensitivity analysis, nonparametric comparisons (Mann-Whitney U for between-group differences at T1 and T2; Wilcoxon signed-rank tests for within-group changes) yielded the same directional effects, providing additional reassurance regarding the consistency of the findings. Mean differences and 95% CIs are reported for all outcomes.

**Table 1.** Pre- and postintervention test performance in ECG^a^ interpretation and text-based ACS^b^ decision-making among participants in a controlled educational intervention comparing FOAMed^c^ videos and podcast with print-based learning materials in Germany in 2024^d^.

	Average of correct answers	
Test component	Print-based media	FOAMed videos and podcast
ECG T1	57	55
ECG T2	60	65.5
Knowledge development	+3%P^e^	+10.5%P
Text questions T1	47	45
Text questions T2	63	68
Knowledge development	+16%P	+23%P

a ECG: electrocardiogram.

b ACS: acute coronary syndrome.

c FOAMed: Free Open Access Medical Education.

d The table presents average correct answers for electrocardiogram and text-based questions at baseline (T1) and postintervention (T2), as well as calculated knowledge development for both intervention groups. Full inferential statistics are provided in Tables S1 and S2 in Multimedia Appendix 3.

e %P denotes percentage points, indicating the absolute difference between two percentages.

Analyses were conducted on all allocated participants with available data (complete-case analysis). Because no participants were lost between T1 and T2, all allocated participants contributed complete paired data. Therefore, the intention-to-treat (ITT) and per-protocol (PP) populations were identical, and the primary analysis effectively reflects both ITT and PP principles.

#### Missing Data

Missing data were assessed at both the participant and item level. No participant-level missingness occurred, as all participants completed the pretest, intervention, and posttest consecutively within a single supervised session (0%). For all ECG interpretation items and clinical decision-making text questions, the response option “I am too unsure” was available and counted as a valid response. In addition, the survey platform (LimeSurvey; LimeSurvey GmbH) required a response before participants could proceed, ensuring complete data for these test items (0% item-level missingness).

For the media evaluation items, overall item-level missingness was 1.2% (24/2043). For the perceived safety ratings, overall missingness was similarly low at 1.2% (6/514). Free-text questions were optional; therefore, non-responses were expected and not considered missing data in the statistical sense.

Because missingness across all quantitative variables was minimal and no inferential analyses required imputation or assumptions about the missing-data mechanism, Missing Completely At Random (MCAR) testing was not applicable.

### Qualitative Analysis (Mayring)

The qualitative component was exploratory and aimed at contextualizing the quantitative findings by capturing participants’ reasons for feeling equally safe or unsafe or less safe after the intervention. The two open-ended survey items were optional and presented only to participants who had indicated unchanged or decreased perceived safety. The exact original German wording was as follows:

- “Beim Erkennen herzkatheter-pflichtiger EKG-Bilder fühle ich mich jetzt gleich (un-)sicher / unsicherer, weil…”
- “Im Umgang mit potenziellen Myokardinfarkt-Patient:innen fühle ich mich jetzt gleich (un-)sicher / unsicherer, weil…”

A total of 46 free-text responses were provided for the question on high-risk ACS ECG interpretation (29 from the print-based group and 17 from the FOAMed group), out of 54 eligible participants. For the question on managing potential myocardial infarction patients, 37 responses were provided (21 print-based and 16 FOAMed), out of 51 eligible participants. As the open-ended items were optional and placed at the end of the survey, the qualitative dataset represents a subset of the quantitative sample.

All responses were transferred verbatim into an Excel matrix and inductively coded following the principles of summary content analysis according to Mayring [33]. The first author conducted the initial coding and assigned content categories, followed by a second step of thematic grouping. To enhance reliability, a didactics-affine internist with experience in qualitative coding reviewed all categories within a coding circle; one minor category adjustment was required, and all discrepancies were resolved by consensus. Irrelevant statements were excluded, and unclear formulations were retained and marked as such. Given the brevity of the free-text responses (typically one short sentence), the qualitative analysis was descriptive in nature and limited in interpretive depth. The responses nevertheless provided sufficient descriptive variation to identify recurring patterns relevant to the study aim. Although the qualitative component consisted of 2 brief free-text questions within a single data-collection wave, thematic saturation was effectively reached, as no new categories emerged during the inductive summary content analysis and all responses clustered within a small set of recurring themes. The qualitative analysis was carried out by 2 emergency-medicine researchers with prior experience in ECG education and qualitative coding. Their professional background and prior familiarity with ACS-related decision-making were explicitly acknowledged during the analytic process, and reflexive discussions were used to limit potential influence on category formation.

The free-text questions were intentionally offered only to participants who reported unchanged or decreased perceived safety, as the aim was to explore reasons for limited confidence gains. This selective sampling strategy aligns with the exploratory purpose of contextualizing heterogeneous changes in perceived safety and was not intended to achieve thematic saturation in the comprehensive qualitative sense.

Methodological integrity was supported through coder consensus, iterative refinement of categories, and grounding all interpretations in verbatim participant statements. Given the exploratory nature and the brevity of responses, the qualitative findings are presented as interpretive insights rather than fully developed thematic conclusions.

### Ethical Considerations

#### Ethical Approval

The study was reviewed and approved by the Ethics Committee of Witten Herdecke University (reference number 139/2023). As the study involved health care providers as participants and assessed the effects of educational interventions on their knowledge and ECG interpretation, without patient involvement or assessment of patient health outcomes, prospective trial registration was not considered applicable according to ICMJE guidance [29].

#### Informed Consent

All participants received written study information and provided informed consent prior to participation. Participation was voluntary, and withdrawal was possible at any time without consequences.

#### Privacy and Confidentiality

All data were collected in a pseudonymized manner using participant-generated individual study codes. No personal identifiers were recorded, and no reidentification key existed within the study team. Although theoretical reidentification would only be possible through external personnel files, the study team had no access to such records. All datasets were stored in accordance with General Data Protection Regulation (GDPR) requirements on secure institutional servers.

#### Compensation

Participants did not receive financial compensation. They were offered the opportunity to enter a raffle for the textbooks used during the study and to attend an optional webinar.

#### Image Use and Identifiability

No identifiable images of participants were collected or included in the paper or supplementary materials. Therefore, no image-related consent was required.

A completed CONSORT-eHEALTH (V1.6) checklist is provided as a checklist file in Checklist 2.

## Results

### Overview

The primary outcome was improvement in ACS-related diagnostic performance from T1 to T2, assessed through 2 coprimary components: ECG interpretation accuracy (high-risk ACS ECG: yes, no, or unsure) and text-based clinical decision-making items (true, false, or unsure). These 2 components were analyzed jointly to capture short-term diagnostic knowledge gain across both intervention groups. Secondary outcomes included subjective confidence in ECG interpretation and ACS management, as well as learning experience ratings. All decision-making results reflect performance in simulated, text-based scenarios rather than real-world clinical actions and therefore represent hypothetical rather than observed clinical behavior.

### Participants and Study Implementation

**Figure 2.**
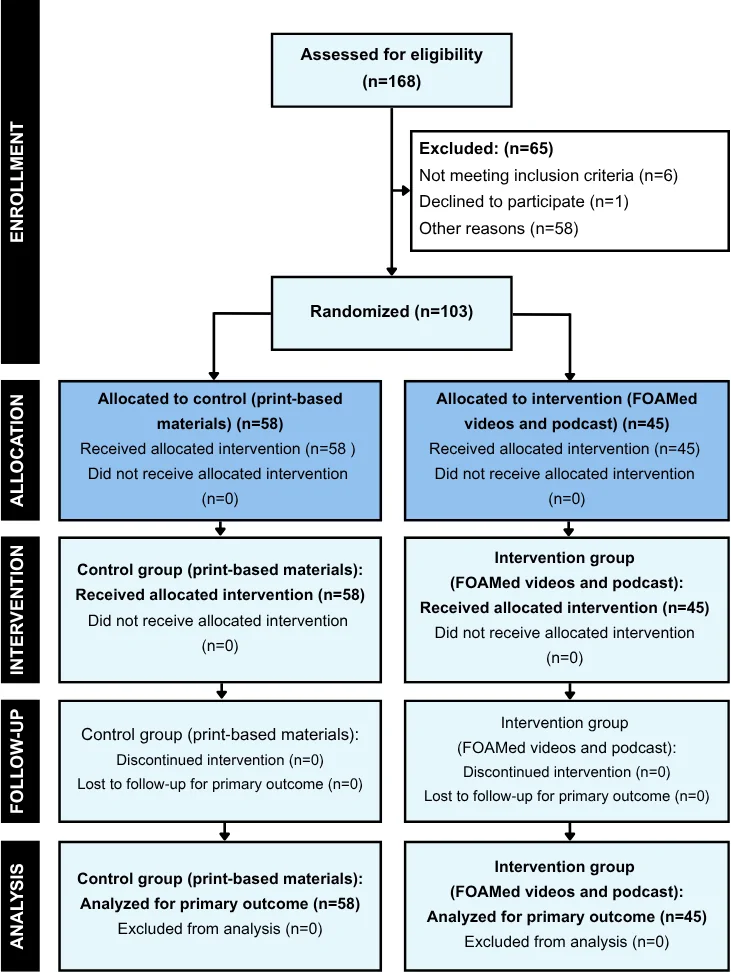
CONSORT (Consolidated Standards of Reporting Trials) 2025 flow diagram of participant progression through the educational intervention. CONSORT 2025 flow diagram showing participant enrollment, allocation, follow-up, and analysis in a controlled educational intervention among emergency care providers in Germany in 2024. FOAMed: Free Open Access Medical Education.

Of the 168 people who originally registered, 103 participants ultimately took part in the study and were allocated. Six of the 65 people who withdrew did not meet the inclusion criteria (including trainees and medical staff not working in emergency medicine), and one doctor declined due to organizational constraints (no free parking). The most significant dropouts were the 58 last-minute cancellations due to staff shortages in the emergency services and clinics, illness (their own or their children’s), and travel problems (train cancellations and car breakdowns). Nine of these 58 people did not show up for their appointments without giving notice. The following CONSORT diagram (Figure 2) visualizes all dropouts. All allocated participants completed both T1 and T2 assessments; therefore, ITT and PP analyses were identical.

The final sample consisted of 71 paramedics and 32 physicians. Three paramedics were also studying medicine. Among the physicians, 21 worked regularly as prehospital emergency physicians, 16 regularly in an emergency department, and 1 in cardiology (multiple responses possible). Further characteristics of the participants are shown in Table 2.

**Table 2.** Sociodemographic and professional characteristics of the participants in a controlled educational intervention comparing FOAMed^a^ videos and podcast with print-based learning materials in Germany in 2024^b^.

Characteristic	Total, n (%)	Intervention group	
		Print-based media, n (%)	FOAMed videos and podcasts, n (%)
Participants	103 (100)	58 (56.3)	45 (43.7)
Gender			
Women	40 (38.8)	25 (62.5)	15 (37.5)
Men	63 (61.2)	33 (52.3)	30 (47.6)
Nonbinary	0 (0)	0 (0)	0 (0)
Age (years)			
18‐25	20 (19.4)	11 (55.0)	9 (45.0)
26‐35	38 (36.9)	21 (55.3)	17 (44.7)
36‐45	29 (28.1)	17 (58.6)	12 (41.4)
46‐55	10 (9.7)	7 (70.0)	3 (30.0)
56‐65	6 (5.8)	2 (33.3)	4 (66.7)
Median age (years)	35	35.1	35
Profession			
Paramedics	71 (68.9)	39 (54.9)	32 (55.1)
Simultaneously medical student	3 (4.2)	3 (100)	0 (0.0)
Medical doctors	32 (31.1)	19 (59.4)	13 (40.6)
Regularly working as a prehospital emergency physician^c^	21 (65.6)	13 (61.9)	8 (38.1)
Regularly working in emergency department^c^	16 (50.0)	10 (62.5)	6 (37.5)
Working in cardiology department^c^	1 (3.1)	0 (0.0)	1 (100.0)
Work experience (years)			
<1	12 (11.65)	6 (50.0)	6 (50.0)
1‐3	24 (23.3)	14 (58.3)	10 (41.7)
4‐7	24	16 (66.7)	8 (33.3)
8‐10	9 (8.7)	6 (66.7)	3 (33.3)
>10	34 (33)	16 (47.0)	18 (53.0)
ECG^d^ interpretations per week (n=101)			
1‐10	52 (50.5)	27 (51.9)	25 (48.1)
11‐25	40 (38.8)	24 (60.0)	16 (40.0)
>25	9 (8.7)	6 (66.7)	3 (33.3)
Sense of safety regarding ECG interpretation			
Safe	9 (8.7)	5 (55.5)	4 (44.5)
Rather safe	47 (45.6)	28 (59.6)	19 (40.4)
Rather unsafe	42 (40.8)	21 (50.0)	21 (50.0)
Unsafe	4 (3.9)	3 (75.0)	1 (25.0)

a FOAMed: Free Open Access Medical Education.

b The table summarizes demographic variables (gender and age), professional background differentiated into paramedics (including 3 simultaneously enrolled medical students) and medical doctors (with multiple possible roles: regularly working as prehospital emergency physicians, in emergency departments, or in cardiology), work experience, weekly electrocardiogram interpretation frequency, and self-reported sense of safety regarding electrocardiogram interpretation, presented for the total sample and stratified by intervention group. The italicized entries indicate subcategories within the same response option. Multiple selections were possible, which is why the totals do not sum to 100%.

c Multiple responses possible.

d ECG: electrocardiogram.

The concealed, nonsystematic allocation resulted in a largely balanced distribution across both intervention groups. Individual peculiarities (eg, all 3 medical students in IG1, the only physician working in cardiology in IG2) had no relevant influence on the group balance.

The allocation of materials was controlled, and there is evidence that participants used the learning resources assigned to them. Protocol adherence was thus ensured. The study design, sample, type of data collected, validity of the evaluation tools, data analysis chosen, and the measured outcomes resulted in a MERSQI score [32] of 16.5 out of a maximum of 18 points. Furthermore, the requirements of the 7-step model of self-directed learning [34] and the methodological recommendations for ECG competence measurement [35] were considered.

### Quantitative Findings

Both intervention groups showed a significant increase in knowledge after the 30-minute learning unit, with this increase being more pronounced in the FOAMed group (IG2; podcast + video) than in the print-based group (IG1; guideline + textbook).

For ECG interpretation, IG2 improved from 55% (T1) to 65.5% (T2), whereas IG1 increased from 57% to 60%. The mixed ANOVA showed a statistically significant interaction between test time (T1 vs T2) and intervention group, assuming sphericity (*F*_1,101_=5.85, *P*=.02, partial η²=0.055, 95% CI 0.001 to 0.158).

For the text-based questions, IG2 improved from 45% (T1) to 68% (T2), while IG1 increased from 47% to 63%. Here, too, there was a statistically significant interaction between test time and intervention group (*F*_1,101_=4.65, *P*=.03, partial η²=0.044, 95% CI 0.000 to 0.142).

The results of this development in knowledge are visualized in Table 1. Detailed inferential statistics (means, SDs, within-group change scores, between-group differences, 95% CIs, *P* values, and effect sizes) are provided in Tables S1 and S2 in Multimedia Appendix 3. CIs are reported to allow readers to assess the precision of the estimates.

IG2 (FOAMed podcast and videos) thus achieved significantly higher learning gains in both areas.

About recognizing ECG patterns requiring emergency cardiac catheterization, 63.6% (28/44) of IG2 participants (FOAMed videos and podcast) reported an increase in their sense of confidence, whereas only 34.5% (20/58) of IG1 participants made this statement. Regarding treatment decisions, 61.4% (27/44) of IG2 participants (FOAMed videos and podcast) reported an increase in their sense of confidence, whereas only 39.3% (22/56) of IG1 participants made this statement.

The FOAMed group (IG2) rated their training unit significantly more positively than the print-based group (IG1). The FOAMed video unit scored highest in the overall evaluation with a mean value of 1.57 (Likert scale: 1=completely agree, 5=completely disagree), followed by the textbooks (mean 2.2), the podcast (mean 2.4), and guidelines (mean 2.6). IG2 also showed the highest willingness to recommend (mean 1.7), while IG1 was more skeptical (mean 2.7). FOAMed videos were also rated as particularly accessible and easy to get used to (mean 4.1 on a scale of 1 = “very difficult to get used to” to 5 = “not at all”), whereas traditional print materials were rated much more critically. In the free-text responses, these were repeatedly described as dry, confusing, or too extensive (eg, “Guidelines are really confusing and there are no illustrative examples. Even in both textbooks, despite many illustrations, there are not enough example ECG” or “For the short time available, textbooks and guidelines are rather unwieldy. I had to read several chapters before I found the answer to a specific question.”) The training content was also very frequently described as very difficult and complicated (eg, “[Topic] is very complicated” or “Too many STEMI equivalents and exceptions”), which was not reflected in the evaluation by the FOAMed group. Table 3 below shows the specific media ratings by the participants.

**Table 3.** Evaluation of learning materials (books, guidelines, videos, and podcast) by participants of a controlled educational intervention comparing FOAMed^a^ videos and podcasts with print-based learning materials in Germany in 2024^b^.

	Books and guidelines (n=58)^c^		FOAMed videos and podcast (n=45)^d^	
	Books (n=58^e^)	Guidelines (n=52^e^)	Videos (n=44^e^)	Podcast (n=30^e^)
Entertaining	2.9	3.4	2.0 (n=43^f^)	2.4 (n=28^f^)
User-friendly	2.8	3.4	1.3	2.4
Comprehensible	2.3	2.9	1.5	2.4
Informative	1.7	1.9	1.4	1.6 (n=29^f^)
Trustworthy	1.55	1.3 (n=51^f^)	1.65	1.9
Mean value of the individual medium	2.25	2.58	1.5	2.41

a FOAMed: Free Open Access Medical Education.

b The table presents Likert-scale ratings (1=completely agree; 5=completely disagree) for perceived qualities of each medium, including entertainment value, user-friendliness, comprehensibility, informativeness, and trustworthiness. Mean values for each individual medium and average values for the 2 intervention units (books and guidelines vs FOAMed videos and podcast) are shown, alongside overall satisfaction, intention to recommend, and perceived need to get used to the medium. Participants only rated the materials they used during the self-learning session, and item-specific sample sizes vary because individual questions could be skipped.

c Mean value of the individual media: books=2.25; guidelines=2.58. Mean value of the intervention unit: books and guidelines=2.4. Overall satisfaction: books and guidelines: mean 2.7, n=57. Intention to recommend: books and guidelines: mean 2.7, n=57. Needing to get used to the medium (mean values): books=3.4; guidelines=2.2.

d Mean value of the individual media: videos=1.5; podcasts=2.41. Mean value of the intervention unit: videos and podcasts=1.9. Overall satisfaction: videos and podcasts: mean 1.8, n=45. Intention to recommend: videos and podcasts: mean 1.7, n=45. Needing to get used to the medium (mean values): videos=4.1; podcasts=2.8.

e Participants only rated the materials they actually used during the self-learning session; unused media were not evaluated.

f Individual item counts differ because participants could skip questions; missing responses were not imputed.

An exploratory subgroup analysis provided no evidence of differential effects, including gender, and was likely underpowered.

### Qualitative Findings

The inductive summary content analysis yielded recurring descriptive categories that explained why some participants did not experience increased perceived safety after the intervention. Across both questions, participants most frequently cited *Zeitmangel, Orientierungsschwierigkeiten, Komplexität des Themas, Ungeeignetes Medium, Fehlendes Feedback, Merk-Schwierigkeiten, Fehlender Praxisbezug, Bereits stabiles Vorwissen*, *and Bereits bestehende Wissensdefizite*. These categories represent structural learning barriers and contextual factors that influenced perceived safety and were descriptive in nature rather than in-depth qualitative themes.

In IG1 (print-based materials), participants most often reported difficulties related to the lack of ECG examples, insufficient feedback, time pressure, unclear structure of the materials, and excessive demands due to the scope or technical language. Overall, IG1 participants perceived the topic as more difficult and cognitively demanding, frequently describing the material as complex, hard to navigate, or overwhelming within the limited time available. Several participants stated that the knowledge they had gained had no effect on their sense of safety, as local guidelines and not individual knowledge determined their clinical decision-making (“There are local guidelines and treatment pathways, so the newly acquired knowledge does not change anything” or “I feel equally (un)certain, as the treatment of ACS patients is determined by an SOP.”).

In IG2 (FOAMed videos + podcasts), didactic shortcomings were mentioned less frequently. Instead, participants more often referred to individual learning problems such as lack of prior knowledge, difficulties in remembering, or insufficient consolidation of newly acquired content. Some participants reported that they would need repeated exposure to the material to feel safer, or that the short duration of the learning unit limited their ability to internalize the content.

Overall, the qualitative findings illustrate why measurable knowledge gains did not uniformly translate into increased perceived safety. They highlight structural barriers (eg, time constraints, medium-related challenges, and lack of feedback) and individual factors (eg, prior knowledge and cognitive load) that help explain heterogeneous changes in perceived safety across groups.

In general, the knowledge gaps in the pre- and posttests affected both newer and established ECG patterns equally, with particularly significant improvements in the identification of de Winter sign and the left main occlusion ECG. STEMI mimics continued to be misinterpreted with striking frequency, for example, confusing pericarditis or hyperkalemia with STEMI equivalents, and frequent incorrect answers to everyday guideline content, such as required additional leads, recommended time intervals until the first 12-lead ECG diagnosis, or acetylsalicylic acid (ASA) dosage.

## Discussion

### Principal Findings

This controlled trial showed that a single 30-minute self-learning session led to clear improvements in ECG interpretation and text-based ACS-related clinical decision-making across all participants. In line with our primary objective, the curated FOAMed multimedia module produced greater short-term learning gains than the selected print-based reference materials under the 30-minute self-learning condition. These findings reflect differences between 2 real-world educational ecosystems rather than a categorical comparison of digital versus print media. Consistent with our secondary aims, FOAMed participants also reported larger increases in perceived confidence when recognizing high-risk ACS ECG patterns and making treatment decisions. Across all analyses, learning benefits were comparable for established and newer high-risk ACS ECG patterns, and no evidence of differential effects was observed; these exploratory analyses were likely underpowered.

The qualitative findings provide interpretive depth to the quantitative results by illustrating why measurable knowledge gains did not uniformly translate into increased perceived safety. Participants frequently described structural barriers such as limited time, difficulty navigating print-based materials, lack of feedback, and the inherent complexity of high-risk ECG patterns. Notably, IG1 participants perceived the topic itself as more difficult and cognitively demanding, suggesting that the medium shaped not only usability but also the perceived complexity of the content. This pattern may align with cognitive load theory [24,25]: some respondents described the print-based materials as difficult to navigate, which could suggest increased extraneous load and reduced cognitive resources for confidence-building. However, this exploratory analysis cannot establish a causal mechanism. In addition, several IG1 participants expressed a more defensive stance toward the relevance of the learning content (eg, reliance on standard operating procedure [SOP] rather than individual diagnostic judgment), which may be consistent with reduced perceived applicability or increased cognitive demands [24,25]. These qualitative insights may offer a possible explanation why confidence improved less consistently in IG1 despite measurable knowledge increases. Together, these responses suggest a possible explanation for the divergent quantitative pattern: some respondents perceived the FOAMed formats as less complex and cognitively demanding, whereas print-based materials were more often described as challenging to navigate. This analysis cannot establish a causal mechanism. Thus, the qualitative component adds explanatory value beyond the quantitative outcomes and helps interpret heterogeneous changes in perceived safety.

Importantly, these improvements in clinical decision-making refer to performance in hypothetical, text-based ACS scenarios rather than real-world clinical behavior, and confidence and sense of safety represent self-reported perceptions rather than objective measures of clinical competence.

Emergency medicine education has increasingly incorporated digital and multimedia-based learning, and our findings are broadly consistent with evidence supporting digital approaches for short-term educational outcomes in healthcare education [26,36,37].

Systematic review evidence suggests that simulation-based training can improve clinical skills acquisition and retention, although the underlying evidence is heterogeneous in quality [36]. Similarly, a systematic review of digital learning of clinical skills found evidence of beneficial effects on learners’ academic performance, while also highlighting heterogeneity across digital modalities and outcomes [37].

In ECG education specifically, asynchronous electronic modules (e-modules) incorporating narrated videos, interactive questions, and feedback have shown potential as an alternative to traditional didactic teaching [38]. Evidence for podcasts is more limited: a recent randomized study reported educational benefits of podcast-based objective structured clinical examination (OSCE) training [39], while systematic review evidence suggests improvements in immediate knowledge retention but remains insufficient to establish broader effects on learning or clinical outcomes [40,41]. These benefits are consistent with cognitive load theory and the cognitive theory of multimedia learning, which posit that audiovisual formats can reduce extraneous load and facilitate the integration of complex visual information such as ECG patterns [24,25]. Our results extend this literature by demonstrating that FOAMed-based self-learning is not only well-received but also more effective than print-based materials for teaching high-risk ACS ECG patterns, a domain known to impose substantial cognitive demands and to be associated with persistent interpretation deficits among both physicians and paramedics [13,18,19]. The observation that print-based materials were perceived as more difficult to navigate is consistent with principles of cognitive load theory, according to which unnecessary demands associated with information presentation may increase extraneous cognitive load and reduce the cognitive resources available for learning [24,25]. Taken together, these findings suggest that curated digital FOAMed resources may better support efficient, practice-relevant learning in emergency care settings than traditional guideline- or textbook-based approaches.

A second key finding of this trial is that a brief, highly structured FOAMed module—combining podcasts and short videos—appeared particularly well suited to support self-directed learning of complex, high-risk ACS ECG patterns in a time-constrained emergency care context. This is consistent with evidence showing that simulation-based and digital learning formats can support clinical skills acquisition and educational outcomes when appropriately designed and aligned with learning objectives [36,37]. Recent work on podcasts in medical education similarly suggests that audio-based resources can promote flexible, self-paced learning and are generally well accepted by learners, although robust evidence on objective learning outcomes remains limited and heterogeneous [39-41]. Our findings extend this literature by demonstrating that FOAMed podcasts and videos can outperform traditional guideline and textbook materials for short, focused upskilling in ACS ECG interpretation and decision-making among practicing emergency care providers. Moreover, the strong learner acceptance and perceived confidence gains observed in the FOAMed are consistent with prior work on self-directed learning and the use of freely accessible educational resources among emergency care professionals [23,42,43]. Such resources may help reduce barriers to continuing education by providing flexible, practice-relevant learning that can be integrated into time-constrained clinical work.

The interprofessional relevance of these findings is underscored by the persistent ECG interpretation deficits across emergency care professions. Prior studies have shown that both physicians and paramedics frequently struggle with ECG interpretation, including recognition of high-risk ACS patterns and STEMI equivalents, with accuracy remaining suboptimal even among experienced clinicians [13,14,18,19]. Recent work further emphasizes the need to recognize a broader spectrum of ischemic ECG patterns beyond classic STEMI criteria, including visually subtle patterns that may be overlooked [10,11]. Against this backdrop, the exploratory subgroup analyses provided no evidence of differential effects, although these analyses were likely underpowered. Within this limitation, the pattern of findings suggests that FOAMed-based learning may offer a scalable and equitable approach to strengthening diagnostic competencies across diverse emergency care roles [23,42,43]. This is particularly relevant given the well-described challenges of maintaining structured continuing education in shift-based systems, where staffing shortages, time constraints, and limited access to formal training opportunities can hinder ongoing skill development [22,44]. By demonstrating that even a brief, self-learning digital intervention can enhance both recognition of high-risk ACS ECG patterns and confidence in clinical decision-making, our findings highlight the potential of FOAMed resources to contribute to interprofessional preparedness, although such implications remain hypothetical. This study also contributes to the broader literature on ECG education by addressing several gaps that have been highlighted in recent research. Prior studies have demonstrated that digital learning formats, including asynchronous e-modules and other structured digital approaches, can improve ECG interpretation skills among medical students and clinicians [38,45]. However, most existing interventions focus on ECG interpretation more broadly, while evidence on teaching newer high-risk ACS and OMI-related ECG patterns remains limited [9,10,27,28]. Recent literature highlights that these patterns are visually subtle, may be underrepresented in conventional ECG teaching, and can be misinterpreted in clinical practice [9-11,27,28]. By incorporating selected high-risk ACS ECG patterns—including guideline-recognized STEMI equivalents and previously described emerging OMI-related patterns—into a controlled design and comparing FOAMed resources directly with traditional print materials, our study extends prior work by demonstrating that digital, case-based multimedia formats can support the teaching of these clinically relevant diagnostic concepts. Furthermore, while earlier studies have often been restricted to single professions or student populations, our interprofessional sample reflects the real-world diversity of emergency care teams and addresses the need for practice-oriented ECG training across professional groups [16,18,19,27]. In this way, the study provides new evidence that curated FOAMed resources can support the acquisition of both established and emerging ischemic ECG patterns across multiple emergency care roles.

Taken together, these findings highlight several contributions to this work. First, the study is innovative in directly comparing a curated FOAMed multimedia module with full-length, deliberately selected print-based reference materials—including the current ACS guideline and a structured textbook chapter—for teaching complex high-risk ACS ECG patterns in an interprofessional emergency care population. Second, it extends existing research by focusing on subtle, emerging ischemic patterns and by evaluating realistic, time-limited self-learning formats rather than isolated digital tools or student samples [27,28,38]. Third, the results contribute new empirical evidence that curated FOAMed resources can improve short-term ECG interpretation and ACS decision-making compared with traditional print-based materials under constrained learning conditions. Finally, while real-world implications remain hypothetical, the findings suggest that concise, high-quality digital modules may represent a feasible and accessible supplement to structured continuing education in shift-based emergency care environments [23,42,43].

### Limitations

This study has several limitations that should be considered when interpreting the findings. First, voluntary participation introduces the possibility of self-selection bias, as individuals with a particular interest in ECG education or digital learning may have been more likely to enroll. Although baseline confidence levels were broadly comparable across groups, recruitment through social media for the final available study slots may have influenced participants’ attitudes toward the media formats. Minor deviations from the intended 1:1 allocation resulted solely from participant no-shows, leaving some preallocated seats unoccupied; this represents a small design limitation. Because the allocation was practically random but not based on a formal computer-generated sequence, the study should be interpreted as using concealed, nonsystematic allocation rather than strict algorithmic randomization. Second, the study focused on a highly specific set of selected high-risk ACS ECG patterns, including guideline-recognized STEMI equivalents and previously described emerging patterns, which limits the generalizability of the results to other clinical topics or educational contexts. Interactive digital formats with feedback functions—which may provide additional educational benefits [46]—were deliberately excluded to maintain feasibility and comparability with print-based materials. Similarly, the decision to contrast freely accessible FOAMed resources with comprehensive but often costly guideline and textbook materials reflected a pragmatic design choice but restricts conclusions about other types of digital or print-based instruction. A further methodological limitation concerns potential allegiance bias, as 2 authors had prior involvement with one FOAMed source; although the selection of materials followed predefined criteria and external verification, prior familiarity cannot be fully excluded as an influencing factor.

Additionally, the fixed 30-minute learning period was perceived by several participants as insufficient for engaging deeply with the material. It is plausible that greater learning gains might have been achieved under more flexible or realistic time conditions. Long-term retention of knowledge could not be assessed within the study timeframe, and follow-up data on sustainability are therefore not yet available. Additionally, although the sample size exceeded 30 participants per group, the target number derived from a priori power analysis was not fully reached. Nevertheless, the achieved sample size provided sufficient data to detect the observed between-group effects, although the study may have been underpowered for smaller effects and subgroup analyses [47-49]. Because no statistical adjustments for multiple comparisons were applied, the risk of type I error is increased, and secondary findings should be interpreted as exploratory.

The qualitative component is subject to additional limitations. The free-text responses were brief (typically one short sentence), optional, and provided only by a subset of participants (54 and 51 eligible respondents), which limits contextual depth and representativeness. As a result, the inductively derived categories reflected descriptive patterns rather than in-depth qualitative themes. Nevertheless, the qualitative findings offer meaningful contextual insights into heterogeneous changes in perceived safety and help explain why knowledge gains did not uniformly translate into increased confidence. They should therefore be interpreted as exploratory, complementary evidence that supports and enriches the quantitative results rather than as stand-alone qualitative themes.

### Conclusion

This controlled trial demonstrates that, under a 30-minute self-learning condition, a curated FOAMed multimedia module can produce greater short-term improvements in ECG interpretation accuracy and text-based ACS decision-making than the full-length print-based reference materials. By directly comparing the tested curated FOAMed module with the tested full-length print-based materials, the study provides novel evidence that the curated FOAMed module was experienced as more accessible within the specific 30-minute condition, rather than supporting general conclusions about multimedia formats. Although the ECG topic is inherently complex, participants experienced the tested FOAMed module as more approachable and less cognitively demanding when delivered through FOAMed formats rather than the print-based texts. The qualitative findings suggest that some print-based materials are more complex and less directly applicable, which may offer a possible explanation for the less consistent confidence gains in the print group despite measurable knowledge improvement. These findings extend existing ECG education research by showing that even emerging ischemic patterns—often underrepresented in guidelines and difficult to teach in conventional formats—can be effectively conveyed through short, focused digital modules. All findings reflect immediate postintervention knowledge and self-reported perceptions only; the study did not assess real-world clinical decision-making, diagnostic performance, patient outcomes, or long-term retention.

The results also highlight the hypothetical potential of FOAMed to complement formal training structures, particularly in shift-based emergency care environments where time constraints and limited access to structured education remain persistent barriers. Integrating high-quality, peer-reviewed digital resources into continuing education may have potential implications, but such implications remain hypothetical and require empirical testing, as this study assessed short-term knowledge gains rather than observed clinical performance. Future research should examine long-term retention, clinical impact, and the optimal combination of digital and interactive learning strategies to further enhance ECG education and emergency cardiovascular care.

## Supplementary material

10.2196/87587 Multimedia Appendix 1 Intervention materials (digital and print resources used in the study).

10.2196/87587 Multimedia Appendix 2 Example test items.

10.2196/87587 Multimedia Appendix 3 Detailed inferential statistics for electrocardiogram interpretation accuracy and text-based acute coronary syndrome clinical decision-making.

10.2196/87587 Checklist 1 Application of the MERSQI checklist to this study.

10.2196/87587 Checklist 2 CONSORT-eHEALTH checklist (V 1.6.x).

## Abbreviations

ACC: American College of Cardiology
ACS: acute coronary syndrome
ASA: acetylsalicylic acid
CONSORT: Consolidated Standards of Reporting Trials
DGK: Deutsche Gesellschaft für Kardiologie
e-module: electronic module
ECG: electrocardiogram
ESC: European Society of Cardiology
FOAMed: Free Open Access Medical Education
GDPR: General Data Protection Regulation
ICMJE: International Committee of Medical Journal Editors
IG1: intervention group 1
IG2: intervention group 2
ITT: intention-to-treat
MCAR: Missing Completely At Random
MERSQI: Medical Education Research Study Quality Instrument
NSTE-ACS: non–ST-segment elevation acute coronary syndrome
OCSE: Objective Structured Clinical Examination
OMI: Occlusion Myocardial Infarction
PP: per-protocol
Q1: baseline questionnaire
Q2: final evaluation questionnaire
SOP: standard operating procedure
STEMI: ST-segment elevation myocardial infarction
T1: pretest
T2: posttest
TIDieR: Template for Intervention Description and Replication
