# Supplementary material for: Teaching Acute Coronary Syndrome High-Risk ECG Interpretation and Clinical Decision-Making Through FOAMed Videos and Podcast Versus Print-Based Materials Among Emergency Care Providers: Randomized Controlled Mixed Methods Trial
Source: JMIR Med Educ. 2026 Sep 15;12:e87587. doi: 10.2196/87587 (PMC13577616; doi:10.2196/87587)
Supplement: Supplementary file 1 [file mededu-v12-e87587-s001.pdf]

Intervention Material

1. PODCAST EPISODE

Title: „titriert“ Hochrisiko EKGs (original German title)

Source: PinUpDocs (FOAMed emergency medicine podcast)

URL: https://pin-up-docs.de/2024/05/18/titriert-hochrisiko-ekgs/

Duration: 27 min 37 s

Format: Embedded audio file within a static PowerPoint-based tablet interface, including navigable timestamps (see figure A1)

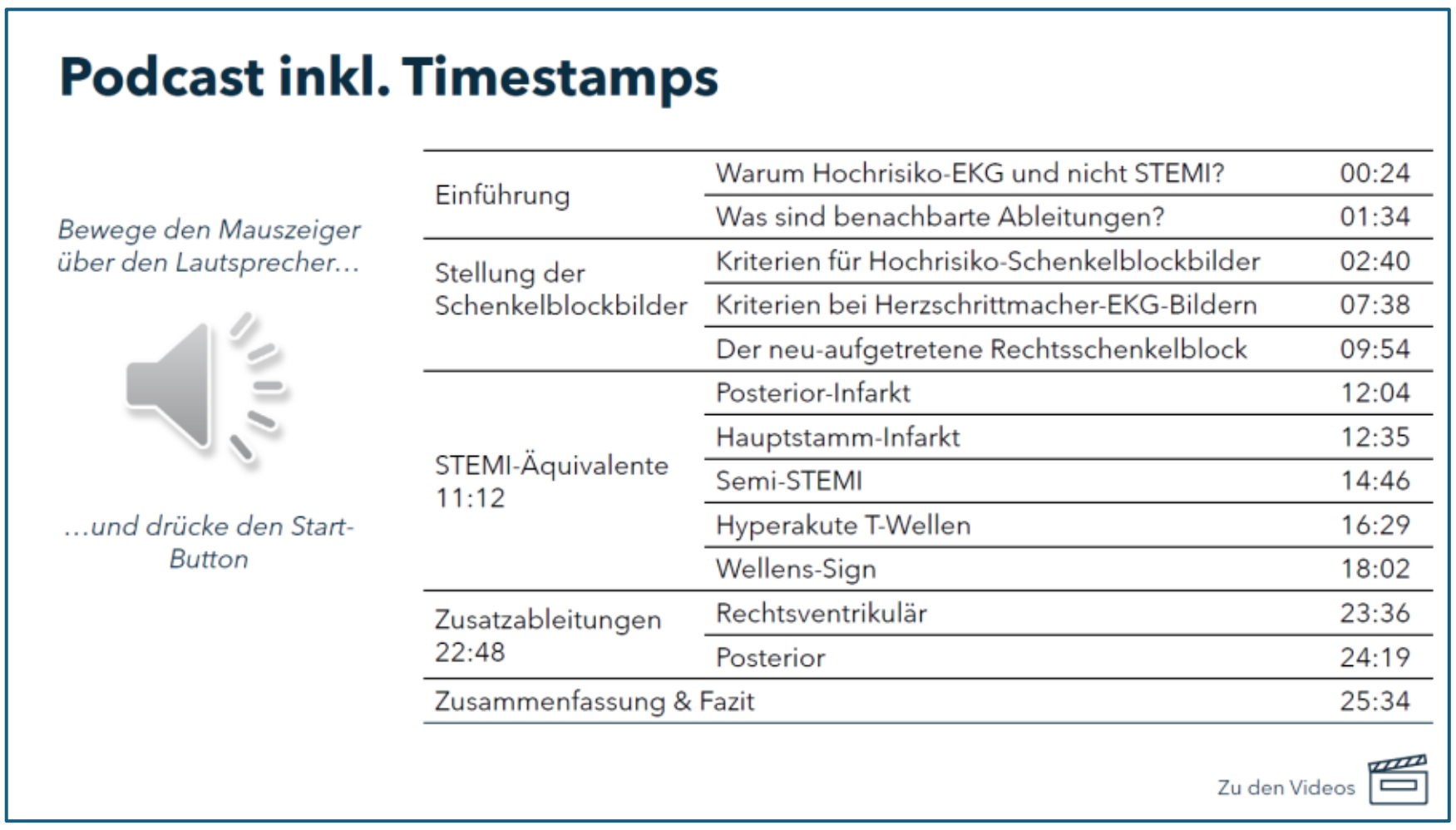

Figure S1: **Screenshot of tablet interface** showing the embedded podcast episode *„titriert“ Hochrisiko EKGs* within the static PowerPoint-based learning module.

2. YOUTUBE VIDEOS

The intervention included nine FOAMed videos originally published on the YouTube channel Nerdfallmedizin (now renamed Notfallmedizin Unplugged). All videos were embedded as MP4 files within the static PowerPoint-based tablet interface used during the study session (see figure A2). Duration includes intro and outro (together approx. ≥1 min).

Playlist (original curation):
https://www.youtube.com/watch?v=dzHC_WIoXTQ&list=PLvF0dhqZCEEsn04eziXchzzZwyhgidC7n

Individual videos

Video 1 - Title: *„Akuter Myokardinfarkt - STEMI (Teil1)“*
URL: https://www.youtube.com/watch?v=dzHC_WIoXTQ
Duration: 14 min 17 s

Video 2 - Title: *„STEMI Teil 2 - Medikamente und mehr“*
URL: https://www.youtube.com/watch?v=uHvbet0lyJc
Duration: 15 min 33 s

Video 3 - Title: *„Myokardinfarkt ohne STEMI? Hochrisiko-EKGs, STEMI-Äquivalente, Schenkelblöcke und mehr…“*
URL: https://www.youtube.com/watch?v=WTt4d33g6UI
Duration: 17 min 51 s

Video 4 - Title: *„EKG-Quickie: Hochlateraler Myokardinfarkt“*
URL: https://www.youtube.com/watch?v=mU1ug939S0Y&t=40s
Duration: 4 min 59 s

Video 5 - Title: *„EKG-Quickie: Posteriorinfarkt!“*
URL: https://www.youtube.com/watch?v=wcK1thnjUgc&t=20s
Duration: 6 min 22 s

Video 6 - Title: *„EKG-Quickie: Bradykardie, Blockbild, Barcelona?!“*
URL: https://www.youtube.com/watch?v=Gf-zpw1T5Ns&t=12s
Duration: 14 min 17 s

Video 7 - Title: *„EKG-Quickie: Winter is coming!“*
URL: https://www.youtube.com/watch?v=QgkAH0PzlW8
Duration: 4 min 58 s

Video 8 - Title: *„EKG-Quickie: Aslanger - ein besonderes Ischämie-EKG!“*
URL: https://www.youtube.com/watch?v=boBZ0PyGDa4&t=5s
Duration: 5 min 53 s

Video 9 - Title: *„EKG-Quickie: Achtung bei Killer-Bs! (oder: Gefährliche Hyperkaliämie rasch im EKG erkennen)“*
URL: https://www.youtube.com/watch?v=k4NuPA9ZKr0&t=7s
Duration: 5 min 27 s

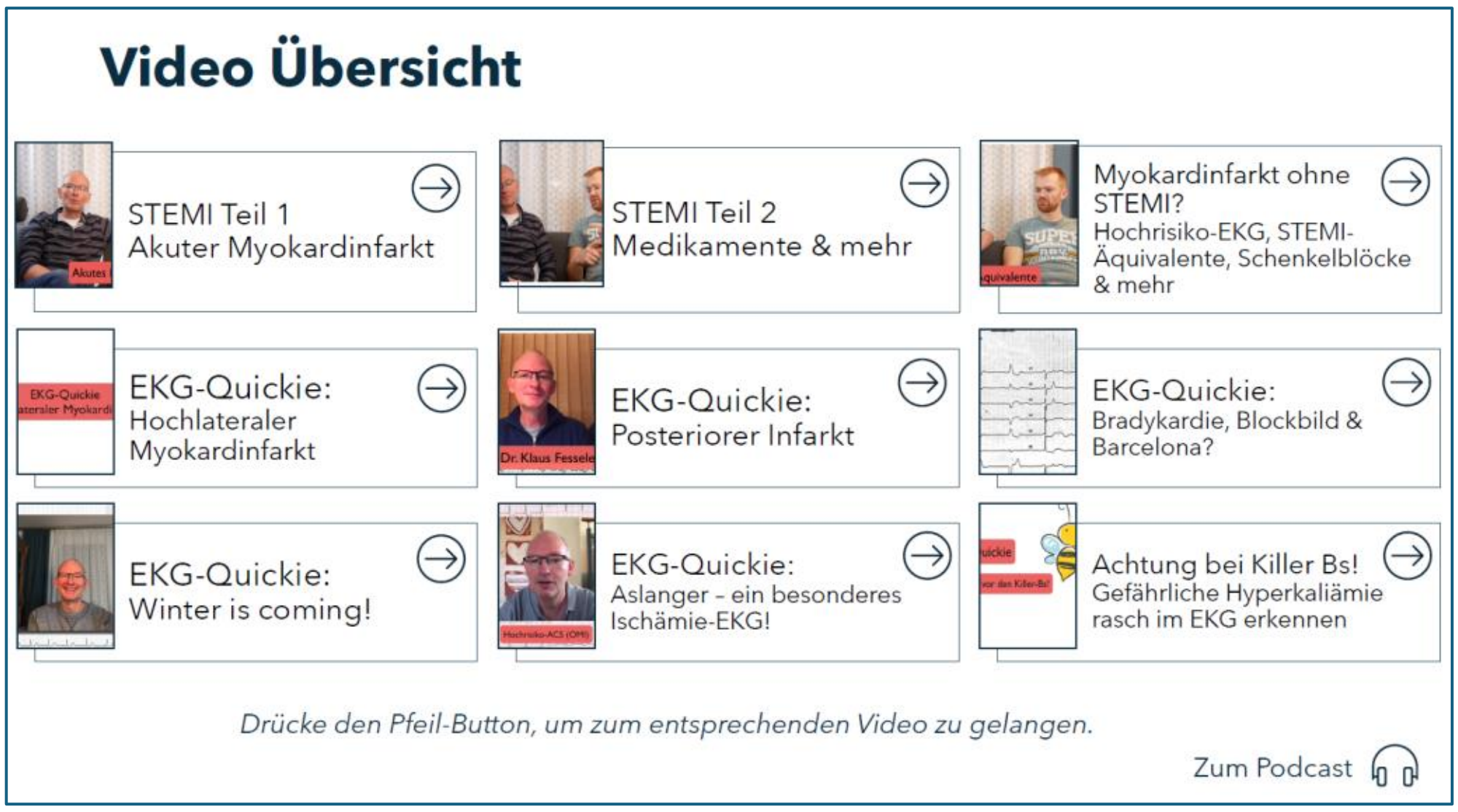

Figure S2: **Screenshot of tablet interface** showing the embedded MP4-files of the nine FOAMed instructional videos within the static PowerPoint-based learning module.
