# Supplementary material for: Teaching Acute Coronary Syndrome High-Risk ECG Interpretation and Clinical Decision-Making Through FOAMed Videos and Podcast Versus Print-Based Materials Among Emergency Care Providers: Randomized Controlled Mixed Methods Trial
Source: JMIR Med Educ. 2026 Sep 15;12:e87587. doi: 10.2196/87587 (PMC13577616; doi:10.2196/87587)
Supplement: Supplementary file 2 [file mededu-v12-e87587-s003.pdf]

Supplementary Table S1. Detailed Inferential Statistics for ECG Interpretation Accuracy (Co-Primary Outcome)

Outcome	Group	n	Mean T1 (%)	SD T1	Mean T2 (%)	SD T2	Δ Within-Group Change (%)	ΔΔ Between-Group Difference (%)
**ECG interpretation accuracy**	FOAMed	45	54.7	13.4	65.6	15.6	+10.9 (95% CI: 5.9 to 15.9)	+8.1 (95% CI: 1.5 to 14.8)
**ECG interpretation accuracy**	Print	58	56.9	16.0	59.7	14.6	+2.8 (95% CI: -1.7 to 7.3)	—

Note. T1 and T2 scores are percentages based on 10 items each. Within-group change was calculated as T2 minus T1. Between-group difference in change represents FOAMed minus Print. Confidence intervals for within-group changes are based on paired change scores; confidence intervals for between-group differences are based on the independent comparison of change scores.

Supplementary Table S2. Detailed Inferential Statistics for Text-Based ACS Clinical Decision-Making (Co-Primary Outcome)

Outcome	Group	n	Mean T1 (%)	SD T1	Mean T2 (%)	SD T2	Δ Within-Group Change (%)	ΔΔ Between-Group Difference (%)
**Text-based ACS decision-making**	FOAMed	45	45.3	15.0	68.4	15.4	+23.1 (95% CI: 18.4 to 27.8)	+6.6 (95% CI: 0.5 to 12.6)
**Text-based ACS decision-making**	Print	58	46.7	15.3	63.3	16.6	+16.6 (95% CI: 12.6 to 20.5)	—

Note. T1 and T2 scores are percentages based on 10 items each. Within-group change was calculated as T2 minus T1. Between-group difference in change represents FOAMed minus Print. Confidence intervals for within-group changes are based on paired change scores; confidence intervals for between-group differences are based on the independent comparison of change scores.
