# Supplementary material for: Teaching Acute Coronary Syndrome High-Risk ECG Interpretation and Clinical Decision-Making Through FOAMed Videos and Podcast Versus Print-Based Materials Among Emergency Care Providers: Randomized Controlled Mixed Methods Trial
Source: JMIR Med Educ. 2026 Sep 15;12:e87587. doi: 10.2196/87587 (PMC13577616; doi:10.2196/87587)
Supplement: Supplementary file 3 [file mededu-v12-e87587-s004.pdf]

MERSQI Checklist

Application of the MERSQI Checklist to the Present Study

Domain	Item		Possible Points	Points Awarded	Justification
Study design	Single-group posttest or cross-sectional study		1	3	Randomized two-group design
	Single-group pre-/posttest study		1,5		
	Two groups, non-randomized		2		
	Two groups, randomized		3		
Sampling	Number of institutions	1	0,5	1,5	Conducted across five institutions
		2	1		
		>2	1,5		
	Response rate	Not applicable		1,5	All enrolled participants completed both assessments within the supervised session
		<50% or not reported	0,5		
		50-74%	1		
		>75%	1,5		
Data type	Participant-reported data		1	3	Primary co-outcomes were objectively scored using predefined correct/incorrect criteria
	Objective measurement		3		
Validity of evaluation instrument	Internal structure	Not applicable		1	Reported: pretesting with target-group representatives who rated item clarity and difficulty, ensuring that items functioned as intended
		Not reported	0		
		Reported	1		
	Inhalt	Not applicable		1	Reported: development of all items based on current guidelines, expert review by a senior emergency cardiologist
		Not reported	0		
		Reported	1		
	Relationships to other variables	Not applicable		1	Reported: subgroup analyses, associations with learning experience, qualitative free-text explanations
		Not reported	0		
		Reported	1		
Data analysis	Appropriateness	Inappropriate for study design/data type	0	1	Mixed ANOVA was appropriate for the paired quantitative dataset and aligned with the prespecified analysis plan
		Appropriate	1		
	Complexity	Descriptive only	1	2	Mixed ANOVA and non-parametric sensitivity checks, representing more complex procedures
		More complex than descriptive	2		
Outcomes	Satisfaction, attitudes, perceptions, opinions, general facts		1	1,5	ECG interpretation and text-based ACS decision-making
	Knowledge / skills		1,5		
	Resulting behavior		2		
	Patient / health outcomes		3		
			**Total Score:**	**16,5** /18	

Reference: Reed DA, Cook DA, Beckman TJ. JAMA 2007;298:1002–1009.
