# Supplementary material for: Teaching Acute Coronary Syndrome High-Risk ECG Interpretation and Clinical Decision-Making Through FOAMed Videos and Podcast Versus Print-Based Materials Among Emergency Care Providers: Randomized Controlled Mixed Methods Trial
Source: JMIR Med Educ. 2026 Sep 15;12:e87587. doi: 10.2196/87587 (PMC13577616; doi:10.2196/87587)
Supplement: Supplementary file 4 [file mededu-v12-e87587-s005.pdf]

CONSORT-EHEALTH (V 1.6.1) - Submission/Publication Form

The CONSORT-EHEALTH checklist is intended for authors of randomized trials evaluating web-based and Internet-based applications/interventions, including mobile interventions, electronic games (incl multiplayer games), social media, certain telehealth applications, and other interactive and/or networked electronic applications. Some of the items (e.g. all subitems under item 5 - description of the intervention) may also be applicable for other study designs.

The goal of the CONSORT EHEALTH checklist and guideline is to be
a) a guide for reporting for authors of RCTs,
b) to form a basis for appraisal of an ehealth trial (in terms of validity)

CONSORT-EHEALTH items/subitems are MANDATORY reporting items for studies published in the Journal of Medical Internet Research and other journals / scientific societies endorsing the checklist.

Items numbered 1., 2., 3., 4a., 4b etc are original CONSORT or CONSORT-NPT (non-pharmacologic treatment) items.
Items with Roman numerals (i., ii, iii, iv etc.) are CONSORT-EHEALTH extensions/ clarifications.

As the CONSORT-EHEALTH checklist is still considered in a formative stage, we would ask that you also RATE ON A SCALE OF 1-5 how important/useful you feel each item is FOR THE PURPOSE OF THE CHECKLIST and reporting guideline (optional).

Mandatory reporting items are marked with a red *.
In the textboxes, either copy & paste the relevant sections from your manuscript into this form - please include any quotes from your manuscript in QUOTATION MARKS,
or answer directly by providing additional information not in the manuscript, or elaborating on why the item was not relevant for this study.

YOUR ANSWERS WILL BE PUBLISHED AS A SUPPLEMENTARY FILE TO YOUR PUBLICATION IN JMIR AND ARE CONSIDERED PART OF YOUR PUBLICATION (IF ACCEPTED).
Please fill in these questions diligently. Information will not be copyedited, so please use proper spelling and grammar, use correct capitalization, and avoid abbreviations.

DO NOT FORGET TO SAVE AS PDF _AND_ CLICK THE SUBMIT BUTTON SO YOUR ANSWERS ARE IN OUR DATABASE !!!

Citation Suggestion (if you append the pdf as Appendix we suggest to cite this paper in the caption):

Eysenbach G, CONSORT-EHEALTH Group
CONSORT-EHEALTH: Improving and Standardizing Evaluation Reports of Web-based and Mobile Health Interventions
J Med Internet Res 2011;13(4):e126
URL: http://www.jmir.org/2011/4/e126/
doi: 10.2196/jmir.1923
PMID: 22209829

wie.turner@web.de Konto wechseln

Nicht freigegeben

Entwurf gespeichert

* Gibt eine erforderliche Frage an

Your name *

First Last

Wiebke Turner

Primary Affiliation (short), City, Country *

University of Toronto, Toronto, Canada

Witten/Herdecke University, Witten, Germany

Your e-mail address *

abc@gmail.com

wiebke.turner@uni-wh.de

Title of your manuscript *

Provide the (draft) title of your manuscript.

Effectiveness of Digital Versus Print-Based Self-Learning Formats for Recognizing ACS High-Risk ECG Patterns: A Randomized Controlled Trial

Name of your App/Software/Intervention *

If there is a short and a long/alternate name, write the short name first and add the long name in brackets.

Curated FOAMed multimedia module (videos +

Evaluated Version (if any)

e.g. "V1", "Release 2017-03-01", "Version 2.0.27913"

Not applicable (curated FOAMed content; no s

Language(s) *

What language is the intervention/app in? If multiple languages are available, separate by comma (e.g. "English, French")

German (videos, podcast, most print materials

URL of your Intervention Website or App

e.g. a direct link to the mobile app on app in appstore (itunes, Google Play), or URL of the website. If the intervention is a DVD or hardware, you can also link to an Amazon page.

Podcast: https://pin-up-docs.de/2024/05/18/titriert-hochrisiko-ekgs/ // Videos: https://www.

Geben Sie eine gültige URL ein.

URL of an image/screenshot (optional)

Meine Antwort

Accessibility *

Can an enduser access the intervention presently?

- (x) access is free and open
- ( ) access only for special usergroups, not open
- ( ) access is open to everyone, but requires payment/subscription/in-app purchases
- ( ) app/intervention no longer accessible
- ( ) Sonstiges:

Primary Medical Indication/Disease/Condition *

e.g. "Stress", "Diabetes", or define the target group in brackets after the condition, e.g. "Autism (Parents of children with)", "Alzheimers (Informal Caregivers of)"

Acute Coronary Syndrome and ECG interpretat

Primary Outcomes measured in trial *

comma-separated list of primary outcomes reported in the trial

ECG interpretation accuracy (ACS high-risk EC

Secondary/other outcomes

Are there any other outcomes the intervention is expected to affect?

Text-based ACS knowledge; subjective confidence in ECG interpretation; subjective confidence in ACS management; learning experience ratings; qualitative explanations for confidence changes; performance in text-based clinical decision-making scenarios

Recommended "Dose" *

What do the instructions for users say on how often the app should be used?

- ○ Approximately Daily
- ○ Approximately Weekly
- ○ Approximately Monthly
- ○ Approximately Yearly
- ○ "as needed"
- ◉ Sonstiges: study-specific: One-time 30-minute self-learning session (no repea

Approx. Percentage of Users (starters) still using the app as recommended after 3 months *

- (selected) unknown / not evaluated
- 0-10%
- 11-20%
- 21-30%
- 31-40%
- 41-50%
- 51-60%
- 61-70%
- 71%-80%
- 81-90%
- 91-100%
- Sonstiges:

Overall, was the app/intervention effective? *

- (●) yes: all primary outcomes were significantly better in intervention group vs control
- ( ) partly: SOME primary outcomes were significantly better in intervention group vs control
- ( ) no statistically significant difference between control and intervention
- ( ) potentially harmful: control was significantly better than intervention in one or more outcomes
- ( ) inconclusive: more research is needed
- ( ) Sonstiges:

Article Preparation Status/Stage *

At which stage in your article preparation are you currently (at the time you fill in this form)

- ( ) not submitted yet - in early draft status
- ( ) not submitted yet - in late draft status, just before submission
- ( ) submitted to a journal but not reviewed yet
- (●) submitted to a journal and after receiving initial reviewer comments
- ( ) submitted to a journal and accepted, but not published yet
- ( ) published
- ( ) Sonstiges:

Journal *

If you already know where you will submit this paper (or if it is already submitted), please provide the journal name (if it is not JMIR, provide the journal name under "other")

- ○ not submitted yet / unclear where I will submit this
- ○ Journal of Medical Internet Research (JMIR)
- ○ JMIR mHealth and UHealth
- ○ JMIR Serious Games
- ○ JMIR Mental Health
- ○ JMIR Public Health
- ○ JMIR Formative Research
- ○ Other JMIR sister journal
- ◉ Sonstiges: JMIR Medical Education

Is this a full powered effectiveness trial or a pilot/feasibility trial? *

- ○ Pilot/feasibility
- ◉ Fully powered

Manuscript tracking number *

If this is a JMIR submission, please provide the manuscript tracking number under "other" (The ms tracking number can be found in the submission acknowledgement email, or when you login as author in JMIR. If the paper is already published in JMIR, then the ms tracking number is the four-digit number at the end of the DOI, to be found at the bottom of each published article in JMIR)

- ○ no ms number (yet) / not (yet) submitted to / published in JMIR
- ◉ Sonstiges: #87587

TITLE AND ABSTRACT

1a) TITLE: Identification as a randomized trial in the title

1a) Does your paper address CONSORT item 1a? *

I.e does the title contain the phrase "Randomized Controlled Trial"? (if not, explain the reason under "other")

- ◉ yes
- ○ Sonstiges:

1a-i) Identify the mode of delivery in the title

Identify the mode of delivery. Preferably use “web-based” and/or “mobile” and/or “electronic game” in the title. Avoid ambiguous terms like “online”, “virtual”, “interactive”. Use “Internet-based” only if Intervention includes non-web-based Internet components (e.g. email), use “computer-based” or “electronic” only if offline products are used. Use “virtual” only in the context of “virtual reality” (3-D worlds). Use “online” only in the context of “online support groups”. Complement or substitute product names with broader terms for the class of products (such as “mobile” or “smart phone” instead of “iphone”), especially if the application runs on different platforms.

	1	2	3	4	5	
subitem not at all important	○	○	○	○	◉	essential

Auswahl löschen

Does your paper address subitem 1a-i? *

Copy and paste relevant sections from manuscript title (include quotes in quotation marks "like this" to indicate direct quotes from your manuscript), or elaborate on this item by providing additional information not in the ms, or briefly explain why the item is not applicable/relevant for your study

Yes. The title explicitly identifies the digital modes of delivery.
The digital components are clearly stated as “FOAMed Videos and Podcast”.
Full title after review: “Teaching ACS High Risk ECG Interpretation and Clinical Decision Making Through FOAMed Videos and Podcast Versus Print-based Materials in Emergency Care Providers: Randomized Controlled Mixed Methods Trial”.

1a-ii) Non-web-based components or important co-interventions in title

Mention non-web-based components or important co-interventions in title, if any (e.g., “with telephone support”).

	1	2	3	4	5	
subitem not at all important	○	○	○	○	◉	essential

Auswahl löschen

Does your paper address subitem 1a-ii?

Copy and paste relevant sections from manuscript title (include quotes in quotation marks "like this" to indicate direct quotes from your manuscript), or elaborate on this item by providing additional information not in the ms, or briefly explain why the item is not applicable/relevant for your study

Yes. The title includes the non-digital comparator (“Print-based Materials”). No additional co-interventions were used.

1a-iii) Primary condition or target group in the title

Mention primary condition or target group in the title, if any (e.g., “for children with Type I Diabetes”) Example: A Web-based and Mobile Intervention with Telephone Support for Children with Type I Diabetes: Randomized Controlled Trial

	1	2	3	4	5	
subitem not at all important	○	○	○	○	◉	essential

Auswahl löschen

Does your paper address subitem 1a-iii? *

Copy and paste relevant sections from manuscript title (include quotes in quotation marks "like this" to indicate direct quotes from your manuscript), or elaborate on this item by providing additional information not in the ms, or briefly explain why the item is not applicable/relevant for your study

Yes. The comparator group ("Print-based Materials") is explicitly mentioned in the title.

1b) ABSTRACT: Structured summary of trial design, methods, results, and conclusions

NPT extension: Description of experimental treatment, comparator, care providers, centers, and blinding status.

1b-i) Key features/functionalities/components of the intervention and comparator in the METHODS section of the ABSTRACT

Mention key features/functionalities/components of the intervention and comparator in the abstract. If possible, also mention theories and principles used for designing the site. Keep in mind the needs of systematic reviewers and indexers by including important synonyms. (Note: Only report in the abstract what the main paper is reporting. If this information is missing from the main body of text, consider adding it)

	1	2	3	4	5	
subitem not at all important	○	○	○	○	◉	essential

Auswahl löschen

Does your paper address subitem 1b-i? *

Copy and paste relevant sections from the manuscript abstract (include quotes in quotation marks "like this" to indicate direct quotes from your manuscript), or elaborate on this item by providing additional information not in the ms, or briefly explain why the item is not applicable/relevant for your study

Yes. The abstract describes the key components of both intervention and comparator.
The digital intervention consisted of a curated FOAMed multimedia module (videos and podcast) designed according to cognitive load theory and FOAMed principles.
The comparator was a print-based self-learning module covering identical content.

1b-ii) Level of human involvement in the METHODS section of the ABSTRACT

Clarify the level of human involvement in the abstract, e.g., use phrases like “fully automated” vs. “therapist/nurse/care provider/physician-assisted” (mention number and expertise of providers involved, if any). (Note: Only report in the abstract what the main paper is reporting. If this information is missing from the main body of text, consider adding it)

	1	2	3	4	5	
subitem not at all important	○	○	○	○	◉	essential

Auswahl löschen

Does your paper address subitem 1b-ii?

Copy and paste relevant sections from the manuscript abstract (include quotes in quotation marks "like this" to indicate direct quotes from your manuscript), or elaborate on this item by providing additional information not in the ms, or briefly explain why the item is not applicable/relevant for your study

Yes. The abstract makes clear that the intervention was a self-directed learning module without provider involvement. Emergency care providers completed a one-time 30-minute self-learning session using either FOAMed videos and podcast or print-based materials. No therapist, tutor, or instructor support was provided.

1b-iii) Open vs. closed, web-based (self-assessment) vs. face-to-face assessments in the METHODS section of the ABSTRACT

Mention how participants were recruited (online vs. offline), e.g., from an open access website or from a clinic or a closed online user group (closed usergroup trial), and clarify if this was a purely web-based trial, or there were face-to-face components (as part of the intervention or for assessment). Clearly say if outcomes were self-assessed through questionnaires (as common in web-based trials). Note: In traditional offline trials, an open trial (open-label trial) is a type of clinical trial in which both the researchers and participants know which treatment is being administered. To avoid confusion, use “blinded” or “unblinded” to indicated the level of blinding instead of “open”, as “open” in web-based trials usually refers to “open access” (i.e. participants can self-enrol). (Note: Only report in the abstract what the main paper is reporting. If this information is missing from the main body of text, consider adding it)

	1	2	3	4	5	
subitem not at all important	○	○	○	○	◉	essential

Auswahl löschen

Does your paper address subitem 1b-iii?

Copy and paste relevant sections from the manuscript abstract (include quotes in quotation marks "like this" to indicate direct quotes from your manuscript), or elaborate on this item by providing additional information not in the ms, or briefly explain why the item is not applicable/relevant for your study

Yes. Participants were recruited digitally via email, while the intervention and assessments were conducted on-site at six locations (one university, two vocational schools, and two hospital conference rooms). The study lead was present to provide standardized instructions, ensure a quiet environment, and supervise adherence to individual work, but no instructional, clinical, or content-related guidance was given. All learning activities and questionnaires were self-administered.

1b-iv) RESULTS section in abstract must contain use data

Report number of participants enrolled/assessed in each group, the use/uptake of the intervention (e.g., attrition/adherence metrics, use over time, number of logins etc.), in addition to primary/secondary outcomes. (Note: Only report in the abstract what the main paper is reporting. If this information is missing from the main body of text, consider adding it)

	1	2	3	4	5	
subitem not at all important	○	○	○	○	●	essential

Auswahl löschen

Does your paper address subitem 1b-iv?

Copy and paste relevant sections from the manuscript abstract (include quotes in quotation marks "like this" to indicate direct quotes from your manuscript), or elaborate on this item by providing additional information not in the ms, or briefly explain why the item is not applicable/relevant for your study

Yes. The abstract reports the number of participants enrolled and analyzed in each group. All participants completed the one-time 30-minute learning session, so no attrition or differential adherence occurred. As the intervention was not web-based, no login or usage log data were applicable.

1b-v) CONCLUSIONS/DISCUSSION in abstract for negative trials

Conclusions/Discussions in abstract for negative trials: Discuss the primary outcome - if the trial is negative (primary outcome not changed), and the intervention was not used, discuss whether negative results are attributable to lack of uptake and discuss reasons. (Note: Only report in the abstract what the main paper is reporting. If this information is missing from the main body of text, consider adding it)

	1	2	3	4	5	
subitem not at all important	○	●	○	○	○	essential

Auswahl löschen

Does your paper address subitem 1b-v?

Copy and paste relevant sections from the manuscript abstract (include quotes in quotation marks "like this" to indicate direct quotes from your manuscript), or elaborate on this item by providing additional information not in the ms, or briefly explain why the item is not applicable/relevant for your study

Not applicable. The trial was not negative; primary and secondary outcomes showed significant improvements. All participants completed the intervention as intended, so no lack of uptake occurred. Therefore, no discussion of negative results or adherence-related explanations was required in the abstract.

INTRODUCTION

2a) In INTRODUCTION: Scientific background and explanation of rationale

2a-i) Problem and the type of system/solution

Describe the problem and the type of system/solution that is object of the study: intended as stand-alone intervention vs. incorporated in broader health care program? Intended for a particular patient population? Goals of the intervention, e.g., being more cost-effective to other interventions, replace or complement other solutions? (Note: Details about the intervention are provided in “Methods” under 5)

	1	2	3	4	5	
subitem not at all important	○	○	○	○	●	essential

Auswahl löschen

Does your paper address subitem 2a-i? *

Copy and paste relevant sections from the manuscript (include quotes in quotation marks "like this" to indicate direct quotes from your manuscript), or elaborate on this item by providing additional information not in the ms, or briefly explain why the item is not applicable/relevant for your study

Yes. The introduction describes the clinical problem (difficulties and high relevance of ACS high-risk ECG interpretation), the target population (emergency care providers), and the type of solution evaluated. The intervention is presented as a stand-alone, self-directed FOAMed multimedia learning module (videos and podcast) intended to improve ECG interpretation and clinical decision making, compared with traditional print-based materials. The rationale includes scalability, accessibility, and the need for effective training formats.

2a-ii) Scientific background, rationale: What is known about the (type of) system

Scientific background, rationale: What is known about the (type of) system that is the object of the study (be sure to discuss the use of similar systems for other conditions/ diagnoses, if appropiate), motivation for the study, i.e. what are the reasons for and what is the context for this specific study, from which stakeholder viewpoint is the study performed, potential impact of findings [2]. Briefly justify the choice of the comparator.

	1	2	3	4	5	
subitem not at all important	○	○	○	○	●	essential

Auswahl löschen

Does your paper address subitem 2a-ii? *

Copy and paste relevant sections from the manuscript (include quotes in quotation marks "like this" to indicate direct quotes from your manuscript), or elaborate on this item by providing additional information not in the ms, or briefly explain why the item is not applicable/relevant for your study

Yes. The introduction outlines the scientific background of FOAMed as a widely used, low-threshold, asynchronous learning approach in medical education. Prior work suggests that multimedia formats such as videos and podcasts can enhance engagement and accessibility, but evidence on their effectiveness for complex diagnostic skills such as ACS high-risk ECG interpretation is limited. The rationale for the study is based on this gap. The comparator (print-based materials) was chosen because it represents a traditional and widely used self-learning format, allowing a content-equivalent comparison.

2b) In INTRODUCTION: Specific objectives or hypotheses

Does your paper address CONSORT subitem 2b? *

Copy and paste relevant sections from the manuscript (include quotes in quotation marks "like this" to indicate direct quotes from your manuscript), or elaborate on this item by providing additional information not in the ms, or briefly explain why the item is not applicable/relevant for your study

Yes. The introduction clearly states the study objectives and hypotheses. The primary objective was to evaluate whether a FOAMed multimedia learning module (videos and podcast) improves ACS high-risk ECG interpretation and clinical decision making compared with print-based self-learning materials. The hypothesis was that the multimedia intervention would lead to superior performance in ECG interpretation and related secondary outcomes.

METHODS

3a) Description of trial design (such as parallel, factorial) including allocation ratio

Does your paper address CONSORT subitem 3a? *

Copy and paste relevant sections from the manuscript (include quotes in quotation marks "like this" to indicate direct quotes from your manuscript), or elaborate on this item by providing additional information not in the ms, or briefly explain why the item is not applicable/relevant for your study

Yes. The manuscript describes the trial as a randomized parallel-group design with a 1:1 allocation ratio. Participants were assigned to either a FOAMed multimedia learning module or print-based self-learning materials, each delivered as a single 30-minute session conducted on-site.

3b) Important changes to methods after trial commencement (such as eligibility criteria), with reasons

Does your paper address CONSORT subitem 3b? *

Copy and paste relevant sections from the manuscript (include quotes in quotation marks "like this" to indicate direct quotes from your manuscript), or elaborate on this item by providing additional information not in the ms, or briefly explain why the item is not applicable/relevant for your study

Not applicable. No changes to the study design, eligibility criteria, intervention, or procedures were made after trial commencement.

3b-i) Bug fixes, Downtimes, Content Changes

Bug fixes, Downtimes, Content Changes: ehealth systems are often dynamic systems. A description of changes to methods therefore also includes important changes made on the intervention or comparator during the trial (e.g., major bug fixes or changes in the functionality or content) (5-iii) and other “unexpected events” that may have influenced study design such as staff changes, system failures/downtimes, etc. [2].

	1	2	3	4	5	
subitem not at all important	◉	○	○	○	○	essential

Auswahl löschen

Does your paper address subitem 3b-i?

Copy and paste relevant sections from the manuscript (include quotes in quotation marks "like this" to indicate direct quotes from your manuscript), or elaborate on this item by providing additional information not in the ms, or briefly explain why the item is not applicable/relevant for your study

Not applicable. The intervention did not involve a dynamic or software-based eHealth system. The multimedia materials (videos and podcast) and print materials were static and did not undergo any changes during the study. No bug fixes, downtimes, or content modifications occurred.

4a) Eligibility criteria for participants

Does your paper address CONSORT subitem 4a? *

Copy and paste relevant sections from the manuscript (include quotes in quotation marks "like this" to indicate direct quotes from your manuscript), or elaborate on this item by providing additional information not in the ms, or briefly explain why the item is not applicable/relevant for your study

Yes. The manuscript clearly specifies the eligibility criteria. As stated in the Participants section: "Participants were paramedics and physicians regularly involved in ACS-related decision-making in emergency care. Inclusion criteria were: age 18–65 years, ≥30 working hours per week, and active clinical responsibility in emergency medicine. Exclusion criteria were: medical students without decision-making authority, trainees, and members of the study team."

4a-i) Computer / Internet literacy

Computer / Internet literacy is often an implicit "de facto" eligibility criterion - this should be explicitly clarified.

	1	2	3	4	5	
subitem not at all important	○	○	●	○	○	essential

Auswahl löschen

Does your paper address subitem 4a-i?

Copy and paste relevant sections from the manuscript (include quotes in quotation marks "like this" to indicate direct quotes from your manuscript), or elaborate on this item by providing additional information not in the ms, or briefly explain why the item is not applicable/relevant for your study

Yes. Although computer or internet literacy was not defined as a formal eligibility criterion, participants needed basic digital skills to navigate the intervention. The multimedia materials were accessed via a pre-configured tablet, requiring touch-based navigation through a prepared PowerPoint interface to start videos and the podcast. Additionally, all questionnaires were administered via LimeSurvey, which required participants to access the survey through a start screen and interact with an internet-based form. Therefore, basic device handling and internet literacy were implicitly required but did not serve as exclusion criteria.

4a-ii) Open vs. closed, web-based vs. face-to-face assessments:

Open vs. closed, web-based vs. face-to-face assessments: Mention how participants were recruited (online vs. offline), e.g., from an open access website or from a clinic, and clarify if this was a purely web-based trial, or there were face-to-face components (as part of the intervention or for assessment), i.e., to what degree got the study team to know the participant. In online-only trials, clarify if participants were quasi-anonymous and whether having multiple identities was possible or whether technical or logistical measures (e.g., cookies, email confirmation, phone calls) were used to detect/prevent these.

	1	2	3	4	5	
subitem not at all important	○	○	○	◉	○	essential

Auswahl löschen

Does your paper address subitem 4a-ii? *

Copy and paste relevant sections from the manuscript (include quotes in quotation marks "like this" to indicate direct quotes from your manuscript), or elaborate on this item by providing additional information not in the ms, or briefly explain why the item is not applicable/relevant for your study

Yes. The manuscript describes how participants were recruited and clarifies that the study was conducted in a closed, in-person setting. As stated: "Recruitment occurred through invitations to hospital departments and emergency services, with remaining slots publicly advertised via social media. Five in-person study events were conducted in North Rhine-Westphalia, Germany, in January 2024." The intervention itself was delivered on-site using tablets with a pre-configured PowerPoint interface for accessing videos and the podcast. All assessments were completed via web-based LimeSurvey questionnaires, which required participants to navigate an internet-based form via a start screen. Participant identity and eligibility were verified in person by the study team.

4a-iii) Information giving during recruitment

Information given during recruitment. Specify how participants were briefed for recruitment and in the informed consent procedures (e.g., publish the informed consent documentation as appendix, see also item X26), as this information may have an effect on user self-selection, user expectation and may also bias results.

	1	2	3	4	5	
subitem not at all important	○	○	○	○	◉	essential

Auswahl löschen

Does your paper address subitem 4a-iii?

Copy and paste relevant sections from the manuscript (include quotes in quotation marks "like this" to indicate direct quotes from your manuscript), or elaborate on this item by providing additional information not in the ms, or briefly explain why the item is not applicable/relevant for your study

Yes. The manuscript describes how participants were informed during recruitment. As stated: “Recruitment occurred through invitations to hospital departments and emergency services, with remaining slots publicly advertised via social media.” The public invitation flyer additionally informed participants about event dates, locations, eligibility criteria, required equipment, and the general study flow. Importantly, the flyer stated: “der konkrete Fortbildungsinhalt bleibt bis zum Termin unbekannt” (“the specific training content remains unknown until the event,” translated by the authors), ensuring that participants could not anticipate the intervention content and thereby minimizing expectation bias. On-site, all participants received standardized written and verbal study information, and written informed consent was obtained before starting the intervention and assessments.

4b) Settings and locations where the data were collected

Does your paper address CONSORT subitem 4b? *

Copy and paste relevant sections from the manuscript (include quotes in quotation marks "like this" to indicate direct quotes from your manuscript), or elaborate on this item by providing additional information not in the ms, or briefly explain why the item is not applicable/relevant for your study

Yes. The manuscript specifies the settings and locations of data collection. As stated: “Five in-person study events were conducted in North Rhine-Westphalia, Germany, in January 2024.” All data were collected on-site during scheduled educational sessions held at hospitals and emergency service facilities. Participants completed the intervention and all assessments in person using study-provided tablets. The public invitation flyer further detailed the event locations (e.g., Bochum, Witten, Herford, Dortmund, Wuppertal), confirming that all study procedures took place in controlled, supervised, face-to-face environments.

4b-i) Report if outcomes were (self-)assessed through online questionnaires

Clearly report if outcomes were (self-)assessed through online questionnaires (as common in web-based trials) or otherwise.

	1	2	3	4	5	
subitem not at all important	○	○	○	○	●	essential

Auswahl löschen

Does your paper address subitem 4b-i? *

Copy and paste relevant sections from the manuscript (include quotes in quotation marks "like this" to indicate direct quotes from your manuscript), or elaborate on this item by providing additional information not in the ms, or briefly explain why the item is not applicable/relevant for your study

Yes. Outcomes were self-assessed through online questionnaires. Participants entered all responses independently using LimeSurvey on study-provided tablets. However, the evaluation of correctness for knowledge items was not performed automatically by the system; scoring and accuracy assessment were conducted retrospectively by the study team.

4b-ii) Report how institutional affiliations are displayed

Report how institutional affiliations are displayed to potential participants [on ehealth media], as affiliations with prestigious hospitals or universities may affect volunteer rates, use, and reactions with regards to an intervention.(Not a required item – describe only if this may bias results)

	1	2	3	4	5	
subitem not at all important	○	○	●	○	○	essential

Auswahl löschen

Does your paper address subitem 4b-ii?

Copy and paste relevant sections from the manuscript (include quotes in quotation marks "like this" to indicate direct quotes from your manuscript), or elaborate on this item by providing additional information not in the ms, or briefly explain why the item is not applicable/relevant for your study

Yes. Institutional affiliations were visible to participants during recruitment. The public invitation flyer displayed the affiliation with "Universität Witten/Herdecke" and listed the supervisors. These affiliations were also visible in the study email address used for registration. However, no institutional branding or logos were shown during the intervention itself, as all learning materials were presented on neutral tablets without university-specific design elements. While institutional affiliation may influence perceived credibility, it was not part of the intervention content and did not differ between study arms.

5) The interventions for each group with sufficient details to allow replication, including how and when they were actually administered

5-i) Mention names, credential, affiliations of the developers, sponsors, and owners

Mention names, credential, affiliations of the developers, sponsors, and owners [6] (if authors/evaluators are owners or developer of the software, this needs to be declared in a "Conflict of interest" section or mentioned elsewhere in the manuscript).

	1	2	3	4	5	
subitem not at all important	○	○	○	●	○	essential

Auswahl löschen

Does your paper address subitem 5-i?

Copy and paste relevant sections from the manuscript (include quotes in quotation marks "like this" to indicate direct quotes from your manuscript), or elaborate on this item by providing additional information not in the ms, or briefly explain why the item is not applicable/relevant for your study

Yes. Educational FOAMed content was selected based on objective, predefined criteria: the FOAMed provider with the highest follower count in the German-speaking emergency medicine community at the time of study conception was chosen. The print-based comparison materials (books) were selected following a preliminary survey among emergency care professionals to identify the most commonly used and recommended resources. Non-financial support was provided by several partners: Apple supplied iPads and keyboards through an Apple Education promotional program aimed at universities; S+K, Thieme and Notfallguru contributed textbooks and book prizes. None of these partners influenced study design, content selection, data collection, analysis, or interpretation. None of the authors or evaluators were owners or developers of proprietary software; all materials were used solely for academic purposes.

5-ii) Describe the history/development process

Describe the history/development process of the application and previous formative evaluations (e.g., focus groups, usability testing), as these will have an impact on adoption/use rates and help with interpreting results.

	1	2	3	4	5	
subitem not at all important	○	○	◉	○	○	essential

Auswahl löschen

Does your paper address subitem 5-ii?

Copy and paste relevant sections from the manuscript (include quotes in quotation marks "like this" to indicate direct quotes from your manuscript), or elaborate on this item by providing additional information not in the ms, or briefly explain why the item is not applicable/relevant for your study

Yes. The intervention did not involve the development of new software; instead, the study materials were created through a structured didactic development process. FOAMed content was selected based on predefined criteria (German-speaking provider with the highest follower count at the time of study conception). The print-based comparison materials were chosen following a preliminary survey among emergency care professionals to identify the most commonly used and recommended textbooks. Clinical guideline excerpts used in the learning materials were selected based on recency and clinical relevance and were reviewed and approved in consultation with a senior cardiology consultant to ensure accuracy and up-to-date alignment with current ACS management standards. The digital learning environment (PowerPoint-based navigation structure with embedded videos and podcast) was iteratively refined and pilot-tested to ensure usability and smooth workflow during the in-person study sessions. No formal usability testing or focus groups were conducted beyond these pilot checks, as the intervention relied on existing, widely used educational materials rather than newly developed software.

5-iii) Revisions and updating

Revisions and updating. Clearly mention the date and/or version number of the application/intervention (and comparator, if applicable) evaluated, or describe whether the intervention underwent major changes during the evaluation process, or whether the development and/or content was “frozen” during the trial. Describe dynamic components such as news feeds or changing content which may have an impact on the replicability of the intervention (for unexpected events see item 3b).

	1	2	3	4	5	
subitem not at all important	○	○	◉	○	○	essential

Auswahl löschen

Does your paper address subitem 5-iii?

Copy and paste relevant sections from the manuscript (include quotes in quotation marks "like this" to indicate direct quotes from your manuscript), or elaborate on this item by providing additional information not in the ms, or briefly explain why the item is not applicable/relevant for your study

Yes. The intervention content was fixed (“frozen”) prior to the start of data collection. All FOAMed materials, textbook excerpts, and guideline sections were selected before the first study session and remained unchanged throughout the entire trial. Clinical guideline excerpts were chosen based on recency and clinical relevance and were reviewed and approved in consultation with a senior cardiology consultant. No updates, revisions, or dynamic content changes occurred during the study period. The comparator materials (textbooks) also remained constant across all sessions.

5-iv) Quality assurance methods

Provide information on quality assurance methods to ensure accuracy and quality of information provided [1], if applicable.

	1	2	3	4	5	
subitem not at all important	○	○	○	○	◉	essential

Auswahl löschen

Does your paper address subitem 5-iv?

Copy and paste relevant sections from the manuscript (include quotes in quotation marks "like this" to indicate direct quotes from your manuscript), or elaborate on this item by providing additional information not in the ms, or briefly explain why the item is not applicable/relevant for your study

Yes. Multiple quality assurance measures were implemented to ensure accuracy and reliability of the educational content. Clinical guideline excerpts were selected based on recency and were reviewed and approved in consultation with a senior cardiology consultant. FOAMed materials were chosen using predefined, objective criteria (largest German-speaking emergency medicine provider by follower count), and print-based comparator materials were selected based on a preliminary survey among emergency care professionals. All digital materials (slides, videos, podcast) were pilot-tested to ensure technical stability and usability on the study tablets. The didactic structure and study workflow were reviewed under academic supervision (Prof. Dr. Jan Ehlers). No automated or dynamic content updates occurred during the study.

5-v) Ensure replicability by publishing the source code, and/or providing screenshots/screen-capture video, and/or providing flowcharts of the algorithms used

Ensure replicability by publishing the source code, and/or providing screenshots/screen-capture video, and/or providing flowcharts of the algorithms used. Replicability (i.e., other researchers should in principle be able to replicate the study) is a hallmark of scientific reporting.

	1	2	3	4	5	
subitem not at all important	○	◉	○	○	○	essential

Auswahl löschen

Does your paper address subitem 5-v?

Copy and paste relevant sections from the manuscript (include quotes in quotation marks "like this" to indicate direct quotes from your manuscript), or elaborate on this item by providing additional information not in the ms, or briefly explain why the item is not applicable/relevant for your study

Not applicable. The intervention did not involve any software development, algorithms, or programmable components. All materials consisted of static educational content (slides, embedded videos, audio files, and printed textbook excerpts). No source code existed, and no dynamic or interactive software elements were used. Replicability is ensured through detailed description of the intervention materials, selection criteria, and study procedures provided in the manuscript.

5-vi) Digital preservation

Digital preservation: Provide the URL of the application, but as the intervention is likely to change or disappear over the course of the years; also make sure the intervention is archived (Internet Archive, webcitation.org, and/or publishing the source code or screenshots/videos alongside the article). As pages behind login screens cannot be archived, consider creating demo pages which are accessible without login.

	1	2	3	4	5	
subitem not at all important	○	◉	○	○	○	essential

Auswahl löschen

Does your paper address subitem 5-vi?

Copy and paste relevant sections from the manuscript (include quotes in quotation marks "like this" to indicate direct quotes from your manuscript), or elaborate on this item by providing additional information not in the ms, or briefly explain why the item is not applicable/relevant for your study

Not applicable. The intervention did not involve a web-based application, login-protected platform, or software that could be archived. All materials consisted of static educational content (slides, embedded videos, audio files, and printed textbook excerpts) delivered locally on study tablets without an online interface. Although no digital preservation of an application was required, all multimedia materials used in the intervention (videos and the podcast episode) were downloaded prior to the study and securely stored to ensure long-term reproducibility of the intervention content. Replicability is ensured through detailed description of all materials and procedures in the manuscript.

5-vii) Access

Access: Describe how participants accessed the application, in what setting/context, if they had to pay (or were paid) or not, whether they had to be a member of specific group. If known, describe how participants obtained “access to the platform and Internet” [1]. To ensure access for editors/reviewers/readers, consider to provide a “backdoor” login account or demo mode for reviewers/readers to explore the application (also important for archiving purposes, see vi).

	1	2	3	4	5	
subitem not at all important	○	○	◉	○	○	essential

Auswahl löschen

Does your paper address subitem 5-vii? *

Copy and paste relevant sections from the manuscript (include quotes in quotation marks "like this" to indicate direct quotes from your manuscript), or elaborate on this item by providing additional information not in the ms, or briefly explain why the item is not applicable/relevant for your study

Yes. Access to the intervention did not require an online platform, login, or payment. All materials were provided locally on study iPads in a supervised setting. Participants accessed the digital learning content directly on the devices without the need for Internet connectivity, registration, or personal accounts. This open, barrier-free access reflects the core principle of FOAMed, which aims to provide freely accessible educational resources. The comparator materials (textbooks) were likewise provided on-site. No external access, membership, or platform credentials were required.

5-viii) Mode of delivery, features/functionalities/components of the intervention and comparator, and the theoretical framework

Describe mode of delivery, features/functionalities/components of the intervention and comparator, and the theoretical framework [6] used to design them (instructional strategy [1], behaviour change techniques, persuasive features, etc., see e.g., [7, 8] for terminology). This includes an in-depth description of the content (including where it is coming from and who developed it) [1]," whether [and how] it is tailored to individual circumstances and allows users to track their progress and receive feedback" [6]. This also includes a description of communication delivery channels and – if computer-mediated communication is a component – whether communication was synchronous or asynchronous [6]. It also includes information on presentation strategies [1], including page design principles, average amount of text on pages, presence of hyperlinks to other resources, etc. [1].

	1	2	3	4	5	
subitem not at all important	○	○	○	●	○	essential

Auswahl löschen

Does your paper address subitem 5-viii? *

Copy and paste relevant sections from the manuscript (include quotes in quotation marks "like this" to indicate direct quotes from your manuscript), or elaborate on this item by providing additional information not in the ms, or briefly explain why the item is not applicable/relevant for your study

Yes. The intervention consisted of a structured, tablet-based digital learning module delivered locally on study iPads. The module included a PowerPoint-based navigation structure with embedded FOAMed videos and one podcast episode, all of which were downloaded in advance and stored offline. The content was not interactive, not tailored to individual learners, and did not include progress tracking or feedback functions. The comparator consisted of printed textbook excerpts selected based on a preliminary survey among emergency care professionals.

The content was curated using predefined criteria: FOAMed materials were selected from the largest German-speaking emergency medicine provider by follower count, and clinical guideline excerpts were chosen based on recency and reviewed with a senior cardiology consultant. The didactic structure followed principles of multimedia learning (segmentation, modality, coherence) and was reviewed under academic supervision (Prof. Dr. Jan Ehlers).

Communication channels were not part of the intervention; no synchronous or asynchronous communication occurred. Presentation strategies included short, segmented video clips, a single podcast episode, and concise slide-based text without hyperlinks or external navigation. All materials were static and identical for all participants.

5-ix) Describe use parameters

Describe use parameters (e.g., intended “doses” and optimal timing for use). Clarify what instructions or recommendations were given to the user, e.g., regarding timing, frequency, heaviness of use, if any, or was the intervention used ad libitum.

	1	2	3	4	5	
subitem not at all important	○	○	◉	○	○	essential

Auswahl löschen

Does your paper address subitem 5-ix?

Copy and paste relevant sections from the manuscript (include quotes in quotation marks "like this" to indicate direct quotes from your manuscript), or elaborate on this item by providing additional information not in the ms, or briefly explain why the item is not applicable/relevant for your study

Yes. The intervention was delivered as a single, time-limited learning session. All participants were instructed to complete a 30-minute learning period, identical for both the digital intervention and the textbook comparator. The duration and timing were fixed and did not allow for self-paced or repeated use. Participants could not access the materials outside the supervised study setting, and no recommendations regarding frequency or additional use were provided. Thus, the intervention was not used ad libitum but followed a standardized, one-time exposure protocol.

5-x) Clarify the level of human involvement

Clarify the level of human involvement (care providers or health professionals, also technical assistance) in the e-intervention or as co-intervention (detail number and expertise of professionals involved, if any, as well as “type of assistance offered, the timing and frequency of the support, how it is initiated, and the medium by which the assistance is delivered”. It may be necessary to distinguish between the level of human involvement required for the trial, and the level of human involvement required for a routine application outside of a RCT setting (discuss under item 21 – generalizability).

	1	2	3	4	5	
subitem not at all important	○	○	○	●	○	essential

Auswahl löschen

Does your paper address subitem 5-x?

Copy and paste relevant sections from the manuscript (include quotes in quotation marks "like this" to indicate direct quotes from your manuscript), or elaborate on this item by providing additional information not in the ms, or briefly explain why the item is not applicable/relevant for your study

Yes. Human involvement consisted of an initial standardized introduction to the study procedure, delivered frontally via projector. A study team member provided the devices or textbooks, explained the workflow, and ensured that all participants started simultaneously. During the learning phase, staff were present only to provide technical support and to maintain quiet, standardized conditions. No instructional, content-related, or pedagogical assistance was offered. Participants completed the 30-minute learning session independently, without interaction with instructors or clinicians. No synchronous or asynchronous communication channels were used. The level of human involvement reflects the intended real-world use, where the digital module is designed for autonomous learning with minimal technical facilitation.

5-xi) Report any prompts/reminders used

Report any prompts/reminders used: Clarify if there were prompts (letters, emails, phone calls, SMS) to use the application, what triggered them, frequency etc. It may be necessary to distinguish between the level of prompts/reminders required for the trial, and the level of prompts/reminders for a routine application outside of a RCT setting (discuss under item 21 – generalizability).

	1	2	3	4	5	
subitem not at all important	(●)	○	○	○	○	essential

Auswahl löschen

Does your paper address subitem 5-xi? *

Copy and paste relevant sections from the manuscript (include quotes in quotation marks "like this" to indicate direct quotes from your manuscript), or elaborate on this item by providing additional information not in the ms, or briefly explain why the item is not applicable/relevant for your study

Not applicable. No prompts or reminders were used. The intervention consisted of a single, supervised 30-minute learning session conducted on-site. Participants did not receive emails, messages, notifications, or any other form of reminder to initiate or continue the intervention. All learning occurred in one session without follow-up prompts, and no automated or human-initiated reminders were part of the study protocol.

5-xii) Describe any co-interventions (incl. training/support)

Describe any co-interventions (incl. training/support): Clearly state any interventions that are provided in addition to the targeted eHealth intervention, as ehealth intervention may not be designed as stand-alone intervention. This includes training sessions and support [1]. It may be necessary to distinguish between the level of training required for the trial, and the level of training for a routine application outside of a RCT setting (discuss under item 21 – generalizability.

	1	2	3	4	5	
subitem not at all important	○	◉	○	○	○	essential

Auswahl löschen

Does your paper address subitem 5-xii? *

Copy and paste relevant sections from the manuscript (include quotes in quotation marks "like this" to indicate direct quotes from your manuscript), or elaborate on this item by providing additional information not in the ms, or briefly explain why the item is not applicable/relevant for your study

Minor co-interventions were present but did not affect the instructional content. All participants received a standardized, frontal introduction to the study procedure via projector. Study staff provided the iPads or textbooks, ensured simultaneous session start, and were available for technical support (e.g., resolving playback or device issues). No instructional, pedagogical, or content-related assistance was provided at any time. No additional materials, communication channels, or follow-up interactions were used. The intervention itself was delivered without any co-interventions that could influence learning outcomes.

6a) Completely defined pre-specified primary and secondary outcome measures, including how and when they were assessed

Does your paper address CONSORT subitem 6a? *

Copy and paste relevant sections from the manuscript (include quotes in quotation marks "like this" to indicate direct quotes from your manuscript), or elaborate on this item by providing additional information not in the ms, or briefly explain why the item is not applicable/relevant for your study

Yes. The primary outcome was improvement in ECG interpretation accuracy from pre-test (T1) to post-test (T2). Participants evaluated a set of standardized ECG cases (ACS high-risk: yes/no/unsure), which were identical at both time points to allow direct comparison. In addition, text-based clinical decision-making questions related to ACS were administered at T1 and T2. Both ECG items and text questions were developed under supervision of a senior emergency cardiologist and presented digitally via LimeSurvey on study tablets.

Secondary outcomes included subjective confidence in ECG interpretation and ACS management, perceived safety, and ratings of the learning experience. These were assessed immediately after the intervention using Likert-scale items within the same LimeSurvey questionnaire.

All outcomes were fully pre-specified, and all assessments occurred on-site immediately before and after the 30-minute learning session. No follow-up measurements were conducted.

6a-i) Online questionnaires: describe if they were validated for online use and apply CHERRIES items to describe how the questionnaires were designed/deployed

If outcomes were obtained through online questionnaires, describe if they were validated for online use and apply CHERRIES items to describe how the questionnaires were designed/deployed [9].

	1	2	3	4	5	
subitem not at all important	○	◉	○	○	○	essential

Auswahl löschen

Does your paper address subitem 6a-i?

Copy and paste relevant sections from manuscript text

Yes. All outcomes were collected using standardized online questionnaires implemented in LimeSurvey, a widely used and validated platform for digital data collection. The questionnaires were deployed locally on study iPads in kiosk mode, ensuring single-entry completion without the possibility of multiple submissions. No IP addresses, cookies, or identifying metadata were stored. All items (ECG cases, text-based ACS questions, and Likert-scale evaluations) were presented in a fixed order, with mandatory responses to prevent missing data.

In accordance with CHERRIES recommendations, participants received a standardized on-site introduction, no incentives were provided, and the survey was accessible only once per participant within the controlled study environment. Data were stored on a secure institutional server.

6a-ii) Describe whether and how “use” (including intensity of use/dosage) was defined/measured/monitored

Describe whether and how “use” (including intensity of use/dosage) was defined/measured/monitored (logins, logfile analysis, etc.). Use/adoption metrics are important process outcomes that should be reported in any ehealth trial.

	1	2	3	4	5	
subitem not at all important	○	○	◉	○	○	essential

Auswahl löschen

Does your paper address subitem 6a-ii?

Copy and paste relevant sections from manuscript text

Yes. “Use” was defined by a fixed 30-minute learning period, but the intensity and pattern of use were self-directed. Participants in the print group could freely navigate the provided textbooks and guidelines, including browsing, re-reading, or skipping sections. Participants in the digital group could move between videos and the podcast, replay segments, or jump within the media files. Learners were not required to consume content continuously and could also use the time for internal rehearsal or reflection.

Because the intervention was delivered offline on study iPads without logins or tracking functions, no digital usage metrics (e.g., timestamps, navigation logs) were recorded. Thus, use was standardized in duration but variable in intensity and sequence, reflecting naturalistic self-directed learning behavior.

6a-iii) Describe whether, how, and when qualitative feedback from participants was obtained

Describe whether, how, and when qualitative feedback from participants was obtained (e.g., through emails, feedback forms, interviews, focus groups).

	1	2	3	4	5	
subitem not at all important	○	○	○	◉	○	essential

Auswahl löschen

Does your paper address subitem 6a-iii?

Copy and paste relevant sections from manuscript text

Yes. Qualitative feedback was obtained through two open-ended questions included in the post-intervention LimeSurvey questionnaire. Participants were asked to explain (1) why they felt more or less confident in recognizing catheter-requiring ECG patterns and (2) why they felt more or less confident in dealing with potential myocardial infarction patients. These free-text responses were analyzed using inductive qualitative content analysis according to Mayring (2015).

The first author conducted the initial coding, and a didactics-affine internist reviewed and refined the emerging categories in a coding circle. Discrepancies were resolved by consensus, resulting in a stable category system. The qualitative items were administered once, immediately after the 30-minute learning session.

6b) Any changes to trial outcomes after the trial commenced, with reasons

Does your paper address CONSORT subitem 6b? *

Copy and paste relevant sections from the manuscript (include quotes in quotation marks "like this" to indicate direct quotes from your manuscript), or elaborate on this item by providing additional information not in the ms, or briefly explain why the item is not applicable/relevant for your study

No changes were made to the pre-specified trial outcomes after the study commenced. All primary and secondary outcomes, including ECG interpretation accuracy, text-based ACS decision items, Likert-scale evaluations, and the two open-ended qualitative questions, were administered exactly as defined in the study protocol. No modifications to outcome definitions, measurement instruments, or assessment time points occurred during the trial.

7a) How sample size was determined

NPT: When applicable, details of whether and how the clustering by care provides or centers was addressed

7a-i) Describe whether and how expected attrition was taken into account when calculating the sample size

Describe whether and how expected attrition was taken into account when calculating the sample size.

	1	2	3	4	5	
subitem not at all important	○	◉	○	○	○	essential

Auswahl löschen

Does your paper address subitem 7a-i?

Copy and paste relevant sections from manuscript title (include quotes in quotation marks "like this" to indicate direct quotes from your manuscript), or elaborate on this item by providing additional information not in the ms, or briefly explain why the item is not applicable/relevant for your study

No expected attrition was included in the sample size calculation because the study consisted of a single supervised on-site session (pre-test, 30-minute intervention, post-test) completed consecutively by all participants. There was no follow-up phase and no risk of participant loss after enrollment. Attrition between recruitment and attendance does not constitute study dropout, as only participants who provided consent and completed the pre-test were enrolled. All randomized participants completed the intervention and both assessments.

7b) When applicable, explanation of any interim analyses and stopping guidelines

Does your paper address CONSORT subitem 7b? *

Copy and paste relevant sections from the manuscript (include quotes in quotation marks "like this" to indicate direct quotes from your manuscript), or elaborate on this item by providing additional information not in the ms, or briefly explain why the item is not applicable/relevant for your study

No interim analyses or stopping guidelines were planned. The study consisted of a single supervised on-site session without follow-up phases or safety-related stopping criteria (see Sample Size section).

8a) Method used to generate the random allocation sequence

NPT: When applicable, how care providers were allocated to each trial group

Does your paper address CONSORT subitem 8a? *

Copy and paste relevant sections from the manuscript (include quotes in quotation marks "like this" to indicate direct quotes from your manuscript), or elaborate on this item by providing additional information not in the ms, or briefly explain why the item is not applicable/relevant for your study

A 1:1 allocation ratio was planned. The allocation sequence (1:1 distribution of intervention materials across pre-configured tablets) was generated before participant arrival by an uninvolved staff member. Randomization was achieved through concealed allocation of these tablets.

8b) Type of randomisation; details of any restriction (such as blocking and block size)

Does your paper address CONSORT subitem 8b? *

Copy and paste relevant sections from the manuscript (include quotes in quotation marks "like this" to indicate direct quotes from your manuscript), or elaborate on this item by providing additional information not in the ms, or briefly explain why the item is not applicable/relevant for your study

The study used simple randomization with an intended 1:1 allocation ratio. No blocking, stratification, or other restrictions were applied. Randomization was implemented through concealed allocation of pre-configured tablets placed non-systematically on tables (see Methods → Randomization).

9) Mechanism used to implement the random allocation sequence (such as sequentially numbered containers), describing any steps taken to conceal the sequence until interventions were assigned

Does your paper address CONSORT subitem 9? *

Copy and paste relevant sections from the manuscript (include quotes in quotation marks "like this" to indicate direct quotes from your manuscript), or elaborate on this item by providing additional information not in the ms, or briefly explain why the item is not applicable/relevant for your study

Randomization was implemented through concealed allocation of pre-configured tablets. Tablets were placed non-systematically on tables, and locked screens prevented visibility of group assignment. Participants and study personnel could not infer allocation prior to unlocking the devices (see Methods → Allocation Concealment).

10) Who generated the random allocation sequence, who enrolled participants, and who assigned participants to interventions

Does your paper address CONSORT subitem 10? *

Copy and paste relevant sections from the manuscript (include quotes in quotation marks "like this" to indicate direct quotes from your manuscript), or elaborate on this item by providing additional information not in the ms, or briefly explain why the item is not applicable/relevant for your study

The random allocation sequence was generated before participant arrival by an uninvolved staff member. Participants were enrolled on-site by attending the study session and generating an individual study code. Assignment to interventions occurred through concealed allocation of pre-configured tablets, which were distributed by an uninformed assistant (see Methods → Randomization → Sequence Generation, Allocation Concealment, and Implementation).

11a) If done, who was blinded after assignment to interventions (for example, participants, care providers, those assessing outcomes) and how

NPT: Whether or not administering co-interventions were blinded to group assignment

11a-i) Specify who was blinded, and who wasn't

Specify who was blinded, and who wasn't. Usually, in web-based trials it is not possible to blind the participants [1, 3] (this should be clearly acknowledged), but it may be possible to blind outcome assessors, those doing data analysis or those administering co-interventions (if any).

	1	2	3	4	5	
subitem not at all important	○	○	○	●	○	essential

Auswahl löschen

Does your paper address subitem 11a-i? *

Copy and paste relevant sections from the manuscript (include quotes in quotation marks "like this" to indicate direct quotes from your manuscript), or elaborate on this item by providing additional information not in the ms, or briefly explain why the item is not applicable/relevant for your study

Blinding of participants and study personnel was not feasible due to the nature of the educational interventions (‘Blinding of participants and study personnel was not feasible due to the nature of the educational interventions.’). Outcome assessment was automated and identical across groups, minimizing assessor bias (‘Outcome assessment was automated and identical across groups, minimizing assessor bias.’). Thus, participants and instructors were not blinded, while outcome assessment was effectively blinded.

11a-ii) Discuss e.g., whether participants knew which intervention was the “intervention of interest” and which one was the “comparator”

Informed consent procedures (4a-ii) can create biases and certain expectations - discuss e.g., whether participants knew which intervention was the “intervention of interest” and which one was the “comparator”.

	1	2	3	4	5	
subitem not at all important	○	○	○	◉	○	essential

Auswahl löschen

Does your paper address subitem 11a-ii?

Copy and paste relevant sections from the manuscript (include quotes in quotation marks "like this" to indicate direct quotes from your manuscript), or elaborate on this item by providing additional information not in the ms, or briefly explain why the item is not applicable/relevant for your study

Participants did not know which intervention was the 'intervention of interest' or which one served as the comparator. The public invitation flyer explicitly stated that 'der konkrete Fortbildungsinhalt bleibt bis zum Termin unbekannt' ('the specific training content remains unknown until the event,' translated by the authors), preventing participants from forming expectations about the content or effectiveness of either format. Although the study was conducted across five sessions within one month and informal information exchange between participants was theoretically possible, any such exchange would have been limited to naming the format (podcast vs. videos) and would not have revealed the actual educational content. Given that both formats were presented as equally valid learning approaches and outcome assessment was automated and objective, the risk of expectation-related bias was minimal.

11b) If relevant, description of the similarity of interventions

(this item is usually not relevant for ehealth trials as it refers to similarity of a placebo or sham intervention to a active medication/intervention)

Does your paper address CONSORT subitem 11b? *

Copy and paste relevant sections from the manuscript (include quotes in quotation marks "like this" to indicate direct quotes from your manuscript), or elaborate on this item by providing additional information not in the ms, or briefly explain why the item is not applicable/relevant for your study

Not applicable. The study compared two active educational interventions and did not include a placebo or sham condition. Therefore, describing similarity between active and placebo interventions is not relevant for this trial.

12a) Statistical methods used to compare groups for primary and secondary outcomes

NPT: When applicable, details of whether and how the clustering by care providers or centers was addressed

Does your paper address CONSORT subitem 12a? *

Copy and paste relevant sections from the manuscript (include quotes in quotation marks "like this" to indicate direct quotes from your manuscript), or elaborate on this item by providing additional information not in the ms, or briefly explain why the item is not applicable/relevant for your study

Yes. Primary and secondary outcomes were analyzed using mixed ANOVAs with the within-subject factor time (pre vs. post) and the between-subject factor group (print vs. FOAMed) ('Primary and secondary outcomes were analyzed using mixed ANOVAs with the within-subject factor time (pre vs. post) and the between-subject factor group (print vs. FOAMed).'). Effect sizes were reported as partial η^2, assumptions were checked prior to analysis, and the significance level was set at $\alpha = .05$ ('Effect sizes were reported as partial η^2…'; 'significance level was set at $\alpha = .05$'). No clustering by centers or providers was present, so NPT-specific adjustments were not applicable.

12a-i) Imputation techniques to deal with attrition / missing values

Imputation techniques to deal with attrition / missing values: Not all participants will use the intervention/comparator as intended and attrition is typically high in ehealth trials. Specify how participants who did not use the application or dropped out from the trial were treated in the statistical analysis (a complete case analysis is strongly discouraged, and simple imputation techniques such as LOCF may also be problematic [4]).

	1	2	3	4	5	
subitem not at all important	(•)	○	○	○	○	essential

Auswahl löschen

Does your paper address subitem 12a-i? *

Copy and paste relevant sections from the manuscript (include quotes in quotation marks "like this" to indicate direct quotes from your manuscript), or elaborate on this item by providing additional information not in the ms, or briefly explain why the item is not applicable/relevant for your study

No imputation techniques were required because there was no attrition and no missing data for primary or secondary outcomes. All randomized participants completed the pre-test, intervention, and post-test consecutively within a single supervised session. There was no participant-level or item-level missingness, so complete-case analysis was identical to the full dataset.

12b) Methods for additional analyses, such as subgroup analyses and adjusted analyses

Does your paper address CONSORT subitem 12b? *

Copy and paste relevant sections from the manuscript (include quotes in quotation marks "like this" to indicate direct quotes from your manuscript), or elaborate on this item by providing additional information not in the ms, or briefly explain why the item is not applicable/relevant for your study

Yes. Several exploratory subgroup analyses were conducted. In addition to examining differences by professional background, further exploratory analyses assessed potential associations with age, gender, and years of professional experience. All subgroup analyses were post hoc, not powered a priori, and were interpreted descriptively. No adjusted analyses were performed.

X26) REB/IRB Approval and Ethical Considerations [recommended as subheading under "Methods"] (not a CONSORT item)

X26-i) Comment on ethics committee approval

	1	2	3	4	5	
subitem not at all important	○	○	○	○	◉	essential

Auswahl löschen

Does your paper address subitem X26-i?

Copy and paste relevant sections from the manuscript (include quotes in quotation marks "like this" to indicate direct quotes from your manuscript), or elaborate on this item by providing additional information not in the ms, or briefly explain why the item is not applicable/relevant for your study

Yes. The study received full ethics approval from the Ethics Committee of Witten/Herdecke University (reference number 139/2023). As stated in the manuscript: 'The study was reviewed and approved by the Ethics Committee of Witten/Herdecke University (reference number 139/2023).' Ethical considerations, including informed consent, data protection, and voluntary participation, are described in the dedicated Ethical Considerations subsection.

x26-ii) Outline informed consent procedures

Outline informed consent procedures e.g., if consent was obtained offline or online (how? Checkbox, etc.?), and what information was provided (see 4a-ii). See [6] for some items to be included in informed consent documents.

	1	2	3	4	5	
subitem not at all important	○	○	○	○	◉	essential

Auswahl löschen

Does your paper address subitem X26-ii?

Copy and paste relevant sections from the manuscript (include quotes in quotation marks "like this" to indicate direct quotes from your manuscript), or elaborate on this item by providing additional information not in the ms, or briefly explain why the item is not applicable/relevant for your study

Yes. Informed consent was obtained in person on-site prior to participation. As stated in the manuscript: 'Informed consent was obtained in person prior to participation.' Participants received standardized written and verbal study information, including study purpose, procedures, data handling, voluntary participation, and the option to withdraw at any time ('Participants received standardized written and verbal study information on-site before participation…'). Consent was documented before the pre-test, and prize draws or certificates were handled independently of performance or participation status.

X26-iii) Safety and security procedures

Safety and security procedures, incl. privacy considerations, and any steps taken to reduce the likelihood or detection of harm (e.g., education and training, availability of a hotline)

	1	2	3	4	5	
subitem not at all important	○	○	○	◉	○	essential

Auswahl löschen

Does your paper address subitem X26-iii?

Copy and paste relevant sections from the manuscript (include quotes in quotation marks "like this" to indicate direct quotes from your manuscript), or elaborate on this item by providing additional information not in the ms, or briefly explain why the item is not applicable/relevant for your study

Yes. The study involved a non-invasive educational intervention, and no harms were expected or observed. Privacy and data security were ensured through pseudonymization and GDPR-compliant data handling. No identifiable images or personal data were collected. All intervention materials were stored locally on study tablets without external data transmission, and participation took place in supervised on-site sessions. Participants could withdraw at any time without consequences.

RESULTS

13a) For each group, the numbers of participants who were randomly assigned, received intended treatment, and were analysed for the primary outcome
NPT: The number of care providers or centers performing the intervention in each group and the number of patients treated by each care provider in each center

Does your paper address CONSORT subitem 13a? *

Copy and paste relevant sections from the manuscript (include quotes in quotation marks "like this" to indicate direct quotes from your manuscript), or elaborate on this item by providing additional information not in the ms, or briefly explain why the item is not applicable/relevant for your study

Yes. As reported in the manuscript, 58 participants were randomized to the print-based group and 45 to the FOAMed group. All randomized participants received the intended intervention and completed both assessments within the supervised session. All 103 participants were analysed for the primary outcome. No clustering by care providers or centers was present, so the NPT extension does not apply.

13b) For each group, losses and exclusions after randomisation, together with reasons

Does your paper address CONSORT subitem 13b? (NOTE: Preferably, this is shown in a CONSORT flow diagram) *

Copy and paste relevant sections from the manuscript (include quotes in quotation marks "like this" to indicate direct quotes from your manuscript), or elaborate on this item by providing additional information not in the ms, or briefly explain why the item is not applicable/relevant for your study

Yes. There were no losses or exclusions after randomisation in either group. All participants who were allocated to a study arm received the intended intervention, completed both assessments within the same supervised session, and were included in the primary outcome analysis. Because the study was conducted in a single on-site session, no dropouts or missing data occurred. The NPT extension does not apply, as no centers or care providers delivered the intervention.

13b-i) Attrition diagram

Strongly recommended: An attrition diagram (e.g., proportion of participants still logging in or using the intervention/comparator in each group plotted over time, similar to a survival curve) or other figures or tables demonstrating usage/dose/engagement.

	1	2	3	4	5	
subitem not at all important	(●)	○	○	○	○	essential

Auswahl löschen

Does your paper address subitem 13b-i?

Copy and paste relevant sections from the manuscript or cite the figure number if applicable (include quotes in quotation marks "like this" to indicate direct quotes from your manuscript), or elaborate on this item by providing additional information not in the ms, or briefly explain why the item is not applicable/relevant for your study

This item is not applicable to the present study. The intervention was delivered in a single supervised on-site session, and all participants completed the full procedure (pre-test, intervention, post-test) without any opportunity for time-based attrition or differential engagement. Because there was no longitudinal use of an app or platform, no logins, and no usage data over time, an attrition or engagement-over-time diagram would not be meaningful for this study.

14a) Dates defining the periods of recruitment and follow-up

Does your paper address CONSORT subitem 14a? *

Copy and paste relevant sections from the manuscript (include quotes in quotation marks "like this" to indicate direct quotes from your manuscript), or elaborate on this item by providing additional information not in the ms, or briefly explain why the item is not applicable/relevant for your study

Yes. Recruitment took place in December 2023, and all study sessions were conducted in January 2024. All participants completed the pre-test, intervention, and post-test within a single supervised on-site session, so follow-up occurred on the same day. No additional follow-up period was planned or conducted.

14a-i) Indicate if critical “secular events” fell into the study period

Indicate if critical “secular events” fell into the study period, e.g., significant changes in Internet resources available or “changes in computer hardware or Internet delivery resources”

	1	2	3	4	5	
subitem not at all important	◉	○	○	○	○	essential

Auswahl löschen

Does your paper address subitem 14a-i?

Copy and paste relevant sections from the manuscript (include quotes in quotation marks "like this" to indicate direct quotes from your manuscript), or elaborate on this item by providing additional information not in the ms, or briefly explain why the item is not applicable/relevant for your study

This item is not applicable. The intervention consisted of static, locally stored educational materials delivered on study tablets without reliance on internet resources or external platforms. The study was conducted within a short, clearly defined period (January 2024), and no relevant secular events or changes in digital infrastructure occurred that could have influenced access, delivery, or outcomes.

14b) Why the trial ended or was stopped (early)

Does your paper address CONSORT subitem 14b? *

Copy and paste relevant sections from the manuscript (include quotes in quotation marks "like this" to indicate direct quotes from your manuscript), or elaborate on this item by providing additional information not in the ms, or briefly explain why the item is not applicable/relevant for your study

The trial was not stopped early. It proceeded exactly as planned, with all study sessions conducted in January 2024 and all randomized participants completing the full procedure. No stopping rules were defined, and no circumstances occurred that would have required early termination.

15) A table showing baseline demographic and clinical characteristics for each group

NPT: When applicable, a description of care providers (case volume, qualification, expertise, etc.) and centers (volume) in each group

Does your paper address CONSORT subitem 15? *

Copy and paste relevant sections from the manuscript (include quotes in quotation marks "like this" to indicate direct quotes from your manuscript), or elaborate on this item by providing additional information not in the ms, or briefly explain why the item is not applicable/relevant for your study

Yes. The manuscript includes a baseline table presenting demographic and educational characteristics for each group, including age, gender, study program, and prior clinical experience (Table 1). These variables were comparable between groups. No clinical characteristics were applicable, as this was an educational intervention with healthy student participants. The NPT extension does not apply because no care providers or centers delivered the intervention.

15-i) Report demographics associated with digital divide issues

In ehealth trials it is particularly important to report demographics associated with digital divide issues, such as age, education, gender, social-economic status, computer/Internet/ ehealth literacy of the participants, if known.

	1	2	3	4	5	
subitem not at all important	○	○	◉	○	○	essential

Auswahl löschen

Does your paper address subitem 15-i? *

Copy and paste relevant sections from the manuscript (include quotes in quotation marks "like this" to indicate direct quotes from your manuscript), or elaborate on this item by providing additional information not in the ms, or briefly explain why the item is not applicable/relevant for your study

Yes. Relevant demographics linked to digital-divide issues (age, gender, profession, work experience) are reported. Because the intervention required only minimal tablet interaction, additional digital-literacy measures were not necessary.

16) For each group, number of participants (denominator) included in each analysis and whether the analysis was by original assigned groups

16-i) Report multiple “denominators” and provide definitions

Report multiple “denominators” and provide definitions: Report N’s (and effect sizes) “across a range of study participation [and use] thresholds” [1], e.g., N exposed, N consented, N used more than x times, N used more than y weeks, N participants “used” the intervention/comparator at specific pre-defined time points of interest (in absolute and relative numbers per group). Always clearly define “use” of the intervention.

	1	2	3	4	5	
subitem not at all important	(•)	( )	( )	( )	( )	essential

Auswahl löschen

Does your paper address subitem 16-i? *

Copy and paste relevant sections from the manuscript (include quotes in quotation marks "like this" to indicate direct quotes from your manuscript), or elaborate on this item by providing additional information not in the ms, or briefly explain why the item is not applicable/relevant for your study

This item is not applicable. The intervention was delivered in a single supervised on-site session, and all randomized participants fully received and completed the intervention. There were no repeated logins, usage thresholds, or longitudinal engagement metrics that would allow reporting multiple denominators or defining different levels of “use.”

16-ii) Primary analysis should be intent-to-treat

Primary analysis should be intent-to-treat, secondary analyses could include comparing only “users”, with the appropriate caveats that this is no longer a randomized sample (see 18-i).

	1	2	3	4	5	
subitem not at all important	( )	( )	( )	(•)	( )	essential

Auswahl löschen

Does your paper address subitem 16-ii?

Copy and paste relevant sections from the manuscript (include quotes in quotation marks "like this" to indicate direct quotes from your manuscript), or elaborate on this item by providing additional information not in the ms, or briefly explain why the item is not applicable/relevant for your study

Yes. All randomized participants received the intervention and were included in the analysis, so the primary analysis corresponds to an intent-to-treat approach. Because there were no losses or exclusions, a separate “users-only” analysis was not applicable.

17a) For each primary and secondary outcome, results for each group, and the estimated effect size and its precision (such as 95% confidence interval)

Does your paper address CONSORT subitem 17a? *

Copy and paste relevant sections from the manuscript (include quotes in quotation marks "like this" to indicate direct quotes from your manuscript), or elaborate on this item by providing additional information not in the ms, or briefly explain why the item is not applicable/relevant for your study

Yes. For all primary and secondary outcomes, group-specific results, effect sizes (partial η^2), and 95% confidence intervals are reported in the Results section. Analyses include mean scores for each group, F-statistics, p-values, and corresponding confidence intervals.

17a-i) Presentation of process outcomes such as metrics of use and intensity of use

In addition to primary/secondary (clinical) outcomes, the presentation of process outcomes such as metrics of use and intensity of use (dose, exposure) and their operational definitions is critical. This does not only refer to metrics of attrition (13-b) (often a binary variable), but also to more continuous exposure metrics such as “average session length”. These must be accompanied by a technical description how a metric like a “session” is defined (e.g., timeout after idle time) [1] (report under item 6a).

	1	2	3	4	5	
subitem not at all important	(●)	( )	( )	( )	( )	essential

Auswahl löschen

Does your paper address subitem 17a-i?

Copy and paste relevant sections from the manuscript (include quotes in quotation marks "like this" to indicate direct quotes from your manuscript), or elaborate on this item by providing additional information not in the ms, or briefly explain why the item is not applicable/relevant for your study

This item is not applicable. The intervention was delivered in a single supervised 30-minute on-site session without logins, repeated sessions, or digital usage tracking. All participants received the full intervention dose, and no metrics of use or intensity of use could be generated.

17b) For binary outcomes, presentation of both absolute and relative effect sizes is recommended

Does your paper address CONSORT subitem 17b? *

Copy and paste relevant sections from the manuscript (include quotes in quotation marks "like this" to indicate direct quotes from your manuscript), or elaborate on this item by providing additional information not in the ms, or briefly explain why the item is not applicable/relevant for your study

Not applicable. Although individual test items were scored as correct vs. incorrect (including “unsure” coded as incorrect), the study outcomes were continuous total scores rather than binary clinical endpoints. Therefore, absolute or relative effect sizes for binary outcomes do not apply.

18) Results of any other analyses performed, including subgroup analyses and adjusted analyses, distinguishing pre-specified from exploratory

Does your paper address CONSORT subitem 18? *

Copy and paste relevant sections from the manuscript (include quotes in quotation marks "like this" to indicate direct quotes from your manuscript), or elaborate on this item by providing additional information not in the ms, or briefly explain why the item is not applicable/relevant for your study

Yes. One exploratory, post-hoc subgroup analysis by gender was conducted and showed no significant differences. No adjusted or additional subgroup or sensitivity analyses were performed.

18-i) Subgroup analysis of comparing only users

A subgroup analysis of comparing only users is not uncommon in ehealth trials, but if done, it must be stressed that this is a self-selected sample and no longer an unbiased sample from a randomized trial (see 16-iii).

	1	2	3	4	5	
subitem not at all important	◉	○	○	○	○	essential

Auswahl löschen

Does your paper address subitem 18-i?

Copy and paste relevant sections from the manuscript (include quotes in quotation marks "like this" to indicate direct quotes from your manuscript), or elaborate on this item by providing additional information not in the ms, or briefly explain why the item is not applicable/relevant for your study

Not applicable. All randomized participants fully used the intervention during the single supervised session, so no separate “users-only” subgroup existed and no such analysis was performed.

19) All important harms or unintended effects in each group
(for specific guidance see CONSORT for harms)

Does your paper address CONSORT subitem 19? *

Copy and paste relevant sections from the manuscript (include quotes in quotation marks "like this" to indicate direct quotes from your manuscript), or elaborate on this item by providing additional information not in the ms, or briefly explain why the item is not applicable/relevant for your study

Not applicable. All participants fully used the intervention during the supervised session. Although participants could choose which specific media within their assigned group to engage with (e.g., video vs. podcast; guideline vs. textbook), no outcome analyses were conducted comparing subgroups based on these within-group media preferences. Therefore, no “users-only” subgroup analysis in the CONSORT-eHEALTH sense was performed.

19-i) Include privacy breaches, technical problems

Include privacy breaches, technical problems. This does not only include physical “harm” to participants, but also incidents such as perceived or real privacy breaches [1], technical problems, and other unexpected/unintended incidents. “Unintended effects” also includes unintended positive effects [2].

	1	2	3	4	5	
subitem not at all important	○	◉	○	○	○	essential

Auswahl löschen

Does your paper address subitem 19-i?

Copy and paste relevant sections from the manuscript (include quotes in quotation marks "like this" to indicate direct quotes from your manuscript), or elaborate on this item by providing additional information not in the ms, or briefly explain why the item is not applicable/relevant for your study

Yes. A few participants initially experienced minor Bluetooth connection issues when pairing their headphones with the tablets, but all problems were resolved before the intervention started. No privacy breaches or other unintended incidents occurred.

19-ii) Include qualitative feedback from participants or observations from staff/researchers

Include qualitative feedback from participants or observations from staff/researchers, if available, on strengths and shortcomings of the application, especially if they point to unintended/unexpected effects or uses. This includes (if available) reasons for why people did or did not use the application as intended by the developers.

	1	2	3	4	5	
subitem not at all important	○	○	◉	○	○	essential

Auswahl löschen

Does your paper address subitem 19-ii?

Copy and paste relevant sections from the manuscript (include quotes in quotation marks "like this" to indicate direct quotes from your manuscript), or elaborate on this item by providing additional information not in the ms, or briefly explain why the item is not applicable/relevant for your study

Yes. Qualitative feedback was collected through two open-ended questions asking participants why they felt more or less confident in recognizing catheter-requiring ECGs and in dealing with potential MI patients. These responses were analyzed using inductive content analysis (Mayring). Media evaluations were collected quantitatively via Likert scales but did not include qualitative comments. No qualitative feedback on technical aspects or reasons for choosing specific media was obtained. Minor initial Bluetooth issues were observed by staff and resolved before the intervention started.

DISCUSSION

22) Interpretation consistent with results, balancing benefits and harms, and considering other relevant evidence

NPT: In addition, take into account the choice of the comparator, lack of or partial blinding, and unequal expertise of care providers or centers in each group

22-i) Restate study questions and summarize the answers suggested by the data, starting with primary outcomes and process outcomes (use)

Restate study questions and summarize the answers suggested by the data, starting with primary outcomes and process outcomes (use).

	1	2	3	4	5	
subitem not at all important	○	○	○	○	◉	essential

Auswahl löschen

Does your paper address subitem 22-i? *

Copy and paste relevant sections from the manuscript (include quotes in quotation marks "like this" to indicate direct quotes from your manuscript), or elaborate on this item by providing additional information not in the ms, or briefly explain why the item is not applicable/relevant for your study

Yes. The Discussion restates the study question and summarizes the findings beginning with the primary outcome. For example, the manuscript states that "a single 30-minute self-learning session led to clear improvements in ECG interpretation" and that FOAMed "produced substantially greater learning gains than the selected print-based reference materials." Secondary outcomes (confidence, ACS knowledge, media evaluations) are also summarized. No process-outcomes applied.

22-ii) Highlight unanswered new questions, suggest future research

Highlight unanswered new questions, suggest future research.

	1	2	3	4	5	
subitem not at all important	○	○	○	○	◉	essential

Auswahl löschen

Does your paper address subitem 22-ii?

Copy and paste relevant sections from the manuscript (include quotes in quotation marks "like this" to indicate direct quotes from your manuscript), or elaborate on this item by providing additional information not in the ms, or briefly explain why the item is not applicable/relevant for your study

Yes. The manuscript explicitly highlights unanswered questions and directions for future research. For example, it notes that "long-term retention of knowledge could not be assessed" and that generalizability to other clinical topics is limited. It also states that conclusions about interactive digital formats cannot be drawn. The Conclusion recommends that "future research should examine long-term retention, clinical impact, and the optimal combination of digital and interactive learning strategies.

20) Trial limitations, addressing sources of potential bias, imprecision, and, if relevant, multiplicity of analyses

20-i) Typical limitations in ehealth trials

Typical limitations in ehealth trials: Participants in ehealth trials are rarely blinded. Ehealth trials often look at a multiplicity of outcomes, increasing risk for a Type I error. Discuss biases due to non-use of the intervention/usability issues, biases through informed consent procedures, unexpected events.

	1	2	3	4	5	
subitem not at all important	○	○	○	○	◉	essential

Auswahl löschen

Does your paper address subitem 20-i? *

Copy and paste relevant sections from the manuscript (include quotes in quotation marks "like this" to indicate direct quotes from your manuscript), or elaborate on this item by providing additional information not in the ms, or briefly explain why the item is not applicable/relevant for your study

Yes. The manuscript addresses several typical eHealth-related limitations. It discusses self-selection bias, potential recruitment bias through FOAMed channels, and explicitly acknowledges the increased risk of Type I error. Usability-related issues were reflected in participants' qualitative feedback (e.g., print materials described as "dry," "confusing," or too extensive). No non-use occurred because all participants used the assigned materials during supervised sessions. No relevant technical problems or unexpected events occurred.

21) Generalisability (external validity, applicability) of the trial findings

NPT: External validity of the trial findings according to the intervention, comparators, patients, and care providers or centers involved in the trial

21-i) Generalizability to other populations

Generalizability to other populations: In particular, discuss generalizability to a general Internet population, outside of a RCT setting, and general patient population, including applicability of the study results for other organizations

	1	2	3	4	5	
subitem not at all important	○	○	○	○	◉	essential

Auswahl löschen

Does your paper address subitem 21-i?

Copy and paste relevant sections from the manuscript (include quotes in quotation marks "like this" to indicate direct quotes from your manuscript), or elaborate on this item by providing additional information not in the ms, or briefly explain why the item is not applicable/relevant for your study

Yes. The manuscript explicitly discusses limits to generalizability. It notes that the study focused on a highly specific set of ACS high-risk ECG patterns, "which limits the generalizability of the results to other clinical topics or educational contexts," and that interactive digital formats were excluded, restricting applicability to other types of digital instruction. Voluntary participation and recruitment through FOAMed channels introduce self-selection bias, limiting transferability to broader Internet or emergency care populations. The Discussion also emphasizes that the study did not assess clinical performance or long-term retention, further constraining external validity.

21-ii) Discuss if there were elements in the RCT that would be different in a routine application setting

Discuss if there were elements in the RCT that would be different in a routine application setting (e.g., prompts/reminders, more human involvement, training sessions or other co-interventions) and what impact the omission of these elements could have on use, adoption, or outcomes if the intervention is applied outside of a RCT setting.

	1	2	3	4	5	
subitem not at all important	○	○	○	○	◉	essential

Auswahl löschen

Does your paper address subitem 21-ii?

Copy and paste relevant sections from the manuscript (include quotes in quotation marks "like this" to indicate direct quotes from your manuscript), or elaborate on this item by providing additional information not in the ms, or briefly explain why the item is not applicable/relevant for your study

Yes. Several elements of the RCT differ from routine use. The manuscript notes that the fixed 30-minute learning period was perceived as insufficient and does not reflect real-world, flexible learning conditions. Materials were strictly allocated and used under supervision ("protocol adherence was thus ensured"), whereas routine use would involve free media choice and variable engagement. Interactive digital formats with feedback were deliberately excluded, limiting applicability to typical FOAMed use outside a trial. The study also did not assess long-term or repeated use, which would be expected in real-world settings. These differences may affect adoption, usability, and learning outcomes in routine application.

OTHER INFORMATION

23) Registration number and name of trial registry

Does your paper address CONSORT subitem 23? *

Copy and paste relevant sections from the manuscript (include quotes in quotation marks "like this" to indicate direct quotes from your manuscript), or elaborate on this item by providing additional information not in the ms, or briefly explain why the item is not applicable/relevant for your study

Yes. The manuscript explicitly states that the study was not prospectively registered because it was a minimal-risk educational intervention without patient data or clinical outcomes and therefore did not fall under mandatory registration requirements.

24) Where the full trial protocol can be accessed, if available

Does your paper address CONSORT subitem 24? *

Cite a Multimedia Appendix, other reference, or copy and paste relevant sections from the manuscript (include quotes in quotation marks "like this" to indicate direct quotes from your manuscript), or elaborate on this item by providing additional information not in the ms, or briefly explain why the item is not applicable/relevant for your study

No. The manuscript does not provide access to a full trial protocol. The Data Availability section states that de-identified datasets, questionnaires, SPSS syntax, and the qualitative coding framework are available upon reasonable request, but no standalone study protocol is publicly accessible. A full protocol was not created for this minimal-risk educational trial.

25) Sources of funding and other support (such as supply of drugs), role of funders

Does your paper address CONSORT subitem 25? *

Copy and paste relevant sections from the manuscript (include quotes in quotation marks "like this" to indicate direct quotes from your manuscript), or elaborate on this item by providing additional information not in the ms, or briefly explain why the item is not applicable/relevant for your study

Yes. The manuscript clearly states that “no financial support was received for this work.” It also specifies non-financial support: “Thieme and S+K provided textbooks… Apple Inc. supported data collection by loaning iPads and keyboards… and the Notfallguru project contributed specialist books for the prize draw.” The manuscript further clarifies that these contributors had no role in study design, data collection, analysis, or interpretation.

X27) Conflicts of Interest (not a CONSORT item)

X27-i) State the relation of the study team towards the system being evaluated

In addition to the usual declaration of interests (financial or otherwise), also state the relation of the study team towards the system being evaluated, i.e., state if the authors/ evaluators are distinct from or identical with the developers/sponsors of the intervention.

	1	2	3	4	5	
subitem not at all important	○	○	○	○	◉	essential

Auswahl löschen

Does your paper address subitem X27-i?

Copy and paste relevant sections from the manuscript (include quotes in quotation marks "like this" to indicate direct quotes from your manuscript), or elaborate on this item by providing additional information not in the ms, or briefly explain why the item is not applicable/relevant for your study

Yes. The manuscript clearly states the relationship between the authors and the FOAMed system evaluated. It notes that “Two authors (MF, KF) had previously contributed to the FOAMed project Nerdfallmedizin, and some of the videos used in this study were originally produced by the Nerdfallmedizin team,” and that WT is part of the broader project but not involved in the specific materials used. The manuscript emphasizes that materials were selected based on objective criteria and independently verified, and that prior involvement “did not influence the selection of materials.” No financial benefits resulted from this engagement.

About the CONSORT EHEALTH checklist

As a result of using this checklist, did you make changes in your manuscript? *

- ○ yes, major changes
- ○ yes, minor changes
- ◉ no

What were the most important changes you made as a result of using this checklist?

Because I had already completely revised the entire manuscript in response to extensive editor and external reviewer comments, most conceptual and structural improvements had already been implemented before I began working through the CONSORT-EHEALTH checklist. As a result, completing the checklist felt more like detailed follow-up work rather than a source of new insights. In retrospect, however, going through the checklist earlier in the process would likely have guided several of the revisions I ultimately made anyway, particularly regarding clarity, transparency, and reporting structure.

How much time did you spend on going through the checklist INCLUDING making changes in your manuscript *

Because the manuscript had already been fully revised in response to extensive editor and reviewer comments, only minimal adjustments were needed during the checklist process; completing it still took approximately four hours, mainly because it required systematically working through each item rather than making substantial changes.

As a result of using this checklist, do you think your manuscript has improved? *

- ○ yes
- ○ no
- ◉ Sonstiges: see above

Would you like to become involved in the CONSORT EHEALTH group?

This would involve for example becoming involved in participating in a workshop and writing an "Explanation and Elaboration" document

- ○ yes
- ◉ no
- ○ Sonstiges:

Auswahl löschen

Any other comments or questions on CONSORT EHEALTH

Meine Antwort

STOP - Save this form as PDF before you click submit
To generate a record that you filled in this form, we recommend to generate a PDF of this page (on a Mac, simply select "print" and then select "print as PDF") before you submit it.

When you submit your (revised) paper to JMIR, please upload the PDF as supplementary file.

Don't worry if some text in the textboxes is cut off, as we still have the complete information in our database. Thank you!

Final step: Click submit !
Click submit so we have your answers in our database!

Senden

Alle Eingaben löschen

Gib niemals Passwörter über Google Formulare weiter.

Dieser Inhalt wurde nicht von Google erstellt und wird von Google auch nicht unterstützt. - Eigentümer dieses Formulars kontaktieren - Nutzungsbedingungen - Datenschutzerklärung

Sieht dieses Formular verdächtig aus? Bericht

Google Formulare
