# Supplementary material for: Teaching Acute Coronary Syndrome High-Risk ECG Interpretation and Clinical Decision-Making Through FOAMed Videos and Podcast Versus Print-Based Materials Among Emergency Care Providers: Randomized Controlled Mixed Methods Trial
Source: JMIR Med Educ. 2026 Sep 15;12:e87587. doi: 10.2196/87587 (PMC13577616; doi:10.2196/87587)
Supplement: Supplementary file 5 [file mededu-v12-e87587-s002.docx]

**1. ECG Example Items**

**Consistent lead‑in:** All ECG items began with the same standardized case vignette, followed immediately by the respective ECG example.

**Patient case:** *time 21:35h*
A 65‑year‑old male presents with 1 hour of pressure‑like chest pain. Relevant history includes long‑standing nicotine use (45 pack‑years). Unless otherwise noted, the last ECG (performed by the general practitioner 2 weeks earlier) showed no pathological findings, in particular no conduction or repolarization abnormalities.

**Question:** Does this ECG represent a STEMI or STEMI‑equivalent requiring immediate catheterization laboratory activation or urgent discussion with an interventional cardiologist? In the prehospital setting: Is there an indication to transport the patient directly to a 24/7 PCI‑capable center?

**Answer options:** Yes / No / Unsure

***Note:*** *Participants received the case vignette in German*

*ECG Example 1:*

Figure S1: **Sinus rhythm with ventricular extrasystole** (severity level 1 of 3)

*ECG Example 2:*

Figure S2: **Aslanger’s sign** (STEMI equivalent; severity level 3 of 3)

**2. Text-based Example Items**

In contrast to the ECG items, these stand‑alone items were presented without a preceding case vignette and assess factual knowledge in a true / false / unsure format.

***Note:*** *The text-based items were presented to participants in German*

**Text-based Item 1** — Severity level 1 of 3

**Stem:** In suspected acute coronary syndrome (ACS), a 12‑lead ECG should be obtained and interpreted no later than 30 minutes after first medical contact.

**Correct answer:** False
**Explanation:** Current guidelines recommend obtaining and interpreting a 12‑lead ECG within 10 minutes of first medical contact.

**Text-based Item 2** — Severity level 3 of 3

**Stem:** In the so‑called De Winter pattern, only leads V2 and V3 are assessed.

**Correct answer:** False
**Explanation:** The De Winter pattern involves precordial leads V1–V6.
